# Comparative efficacy of non-pharmacological interventions for amnestic mild cognitive impairment: a network meta-analysis

**DOI:** 10.3389/fnagi.2026.1878590

**Published:** 2026-10-02

**Authors:** Baofang Zhang, Xuanling Li, Qian Zhao, Shuqun Zhang, Yuquan Bai, Youlin Duan, Caien Yu, Chenlin Sui, Aochen Yang, Lianwei Xu

**Affiliations:** 1 Department of Gynecology, Longhua Hospital, Shanghai University of Traditional Chinese Medicine, Shanghai, China; 2 College of Computer Science and Technology, Nanjing University of Aeronautics and Astronautics, Nanjing, China

**Keywords:** acupuncture, amnestic mild cognitive impairment, moxibustion, network meta-analysis, non-pharmacological intervention, traditional Chinese medicine

## Abstract

**Background:**

Amnestic mild cognitive impairment (aMCI) represents a critical prodromal stage of Alzheimer's disease, with a prevalence of approximately 10.03% and an annual conversion rate to dementia of 5%–10%. Non-pharmacological interventions, including acupuncture, moxibustion, cognitive therapies, repetitive transcranial magnetic stimulation, and Tai Chi, are considered potentially effective therapeutic approaches. However, comparative efficacy among these interventions remains elusive.

**Methods:**

PubMed, Embase, Cochrane Library, Web of Science, CNKI, Wanfang, and VIP databases were systematically searched from inception to March 2026. Randomized controlled trials comparing two or more non-pharmacological interventions for aMCI were included. The RoB2 was used to assess methodological quality. A frequentist network meta-analysis was conducted with changes in cognitive function scores as the primary outcome, measured by the Mini-Mental State Examination (MMSE) and Montreal Cognitive Assessment (MoCA). Statistical analyses were performed using R software (version 4.4.3) with the netmeta package. Mean differences (MD) and 95% confidence intervals (CI) were calculated. The surface under the cumulative ranking curve (SUCRA) was used to rank interventions. Consistency was evaluated using the node-splitting method, publication bias was assessed with funnel plots, and robustness was examined through sensitivity analysis.

**Results:**

A total of 32 randomized controlled trials were included, of which 22 reported MMSE scores and 21 reported MoCA scores. Network meta-analysis demonstrated that manual acupuncture combined with moxibustion ranked first for improving MMSE scores (SUCRA = 87.9%), showing significant superiority over usual care (MD = 1.65, 95% CI: 0.01 to 3.29), followed by Tai Chi (SUCRA = 76.7%) and electroacupuncture (SUCRA = 71.7%). For MoCA score improvement, manual acupuncture combined with moxibustion also ranked first (SUCRA = 95.3%), with manual acupuncture (SUCRA = 71.5%) and moxibustion (SUCRA = 65.9%) ranking second and third, respectively. Both outcome measures yielded consistent results, indicating that traditional Chinese medicine-based non-pharmacological interventions demonstrated favorable efficacy with higher rankings. However, absolute effect sizes were modest, and the top ranking of the combined intervention was tentative given local inconsistency and the small number of informing trials.

**Conclusions:**

Non-pharmacological interventions confer modest beneficial effects on cognitive function in patients with amnestic mild cognitive impairment, and traditional Chinese medicine-based modalities consistently occupied the top ranks for both outcomes. Manual acupuncture combined with moxibustion ranked highest; however, this ranking is tentative given local inconsistency and the small number of informing trials, and the certainty of evidence was limited by the methodological quality of the included studies. Given their favorable safety profiles, these interventions are best positioned as adjunctive therapies. Large-scale, rigorously designed randomized controlled trials, including head-to-head comparisons of combined vs. single-modality interventions, are warranted to substantiate these findings.

**Systematic review registration:**

PROSPERO: https://www.crd.york.ac.uk/prospero/display_record.php?ID=CRD420251007806, identifier: CRD420251007806.

## 1 Introduction

Mild cognitive impairment (MCI) represents a critical prodromal stage of Alzheimer's disease (AD). Based on the presence or absence of memory impairment, MCI can be classified into two subtypes: amnestic MCI (aMCI) and non-amnestic MCI (naMCI). Epidemiological data indicate that aMCI accounts for the highest proportion among MCI subtypes, with a prevalence of approximately 10.03% (Bai et al., 2022), and its incidence is significantly higher in economically underdeveloped regions compared to developed areas (Liu et al., 2021). From a pathological perspective, aMCI demonstrates the closest association with AD pathology. Studies have shown that aMCI patients exhibit more severe β-amyloid deposition than other MCI subtypes (Yeung et al., 2022), which explains why aMCI has the highest conversion rate to AD (Edmonds et al., 2024), with an annual conversion rate of 5%–10% (Lombardi et al., 2020). Fortunately, aMCI is potentially reversible (McGirr et al., 2022), and some patients can restore normal cognitive function through early intervention. Existing evidence confirms that non-pharmacological approaches, including cognitive training, lifestyle interventions, transcranial electromagnetic stimulation, acupuncture, and Tai Chi, can effectively improve cognitive function in patients with aMCI. Given the pivotal role of aMCI in AD progression and its amenability to intervention, early identification and management of aMCI have become essential strategies for dementia prevention in clinical practice.

Currently, no pharmacological interventions have been approved globally for MCI. Although certain pharmacological agents, such as sodium benzoate (Lin et al., 2025) and levetiracetam extended-release formulations (Bakker et al., 2024), have demonstrated modest improvements in overall cognitive function in clinical trials, these investigations remain experimental and have not yet received regulatory approval for widespread clinical use. Consequently, non-pharmacological interventions have emerged as critical strategies for delaying cognitive decline in MCI.

Among existing non-pharmacological therapies, cognitive behavioral therapy enhances cognitive function through structured training that promotes neuroplasticity, strengthens synaptic connectivity, and facilitates neural network reorganization (Dai et al., 2023). Non-invasive brain stimulation techniques, including repetitive transcranial magnetic stimulation and transcranial direct current stimulation, can modulate cortical excitability, enhance neuroplasticity and functional connectivity, and may exert anti-neuroinflammatory effects (Cirillo et al., 2023; Wu et al., 2022). Aerobic exercise improves cognitive function by promoting hippocampal neurogenesis, increasing cerebral blood flow, and reducing vascular resistance (Lan et al., 2018; Corkery, 2022). Virtual reality activates and reorganizes neural networks through immersive, multi-sensory interactive environments (Moulaei et al., 2024). Concurrently, traditional Chinese medicine therapies offer distinct advantages. Acupuncture has been demonstrated to improve cognitive function through multiple mechanisms, including modulation of brain network connectivity (Tan et al., 2017), reduction of β-amyloid deposition and tau hyperphosphorylation, attenuation of oxidative stress with enhanced ATP and antioxidant enzyme activity (Wang et al., 2025), and regulation of peripheral immune function and neuroinflammation (Zhi et al., 2021). Moxibustion exerts beneficial effects through enhanced cerebral energy metabolism (Ha et al., 2020) and improved intestinal flora homeostasis (Jia et al., 2022). Multiple clinical studies have validated that traditional Chinese medicine exercises, such as Baduanjin and Tai Chi, which integrate physical movements, breathing regulation, and meditation, can improve global cognitive function, executive function, and memory in patients with cognitive impairment (Yao et al., 2023). These interventions not only demonstrate significant clinical efficacy but are also characterized by operational simplicity and cost-effectiveness. However, existing studies are predominantly limited to comparisons between single interventions and placebo or no-treatment controls, with a paucity of head-to-head comparisons among different therapeutic modalities. This evidence gap precludes clinicians from making informed, evidence-based decisions regarding optimal treatment selection.

Conventional pairwise meta-analytic approaches are restricted to direct comparisons between two interventions and are incapable of establishing comprehensive efficacy hierarchies in multi-intervention contexts. Furthermore, the majority of existing meta-analyzes have not explicitly stratified MCI subtypes. Given the relative scarcity of head-to-head randomized controlled trials of non-pharmacological interventions for aMCI, network meta-analysis (NMA) offers a methodological advantage by constructing a connected network of interventions to integrate direct and indirect evidence, thereby enabling simultaneous comparison and hierarchical ranking of multiple treatments. This study specifically focuses on the aMCI subtype and employs NMA to comprehensively evaluate three categories of non-pharmacological interventions: cognitive training, non-invasive brain stimulation, and traditional Chinese medicine therapies. Using Mini-Mental State Examination (MMSE) and Montreal Cognitive Assessment (MoCA) scores as primary outcome measures, this investigation aims to elucidate the differential effects of various interventions on cognitive function in aMCI.

## 2 Materials and methods

### 2.1 Protocol and registration

This study protocol was registered with PROSPERO (registration number: CRD420251007806). The reporting process strictly followed the Preferred Reporting Items for Systematic Reviews and Meta-Analyses (PRISMA) 2020 statement to ensure transparency and standardization of the research.

### 2.2 Eligibility criteria

The eligibility criteria were designed in accordance with the PICOS framework. Studies meeting the following criteria were included: (1) Population (P): adults diagnosed with aMCI according to internationally recognized diagnostic criteria, without restriction on age, sex, or setting; (2) Interventions (I): any non-pharmacological intervention (e.g., manual acupuncture, moxibustion, electroacupuncture, Tai Chi, cognitive interventions, or non-invasive brain stimulation); (3) Comparators (C): another eligible non-pharmacological intervention, or a control condition (active control, placebo/sham control, treatment as usual, or no intervention; defined below); (4) Outcomes (O): change in MMSE and/or MoCA scores; (5) Study design (S): randomized controlled trials.

Studies were excluded if they met any of the following criteria: (1) secondary cognitive impairment (such as post-stroke MCI, traumatic MCI, or chemotherapy-related cognitive impairment); (2) aMCI subtype not specified; (3) non-RCT study designs (such as systematic reviews, narrative reviews, theoretical discussions, case reports, and animal studies); (4) duplicate publications or studies with overlapping data; (5) incomplete data or inability to extract data; (6) interventions reported in only a single study.

Eligible comparator conditions were classified into four categories. Active control (AC): structured non-specific activities matched for contact time and attention (e.g., stretching exercise, health education, or general cognitive stimulation). Placebo control (PC): sham procedures mimicking the active intervention without its specific components (e.g., sham acupuncture, sham rTMS). Treatment as usual (TAU): routine care, including stable conventional medication where applicable, without any study-specific intervention. No intervention (NI): waiting-list or no-treatment conditions.

### 2.3 Search strategy

We systematically searched PubMed, Embase, Cochrane Library, Web of Science, and three Chinese databases including China National Knowledge Infrastructure (CNKI), VIP Database, and Wanfang Database, from inception to March 2026. The search strategy was constructed using a combination of Medical Subject Headings (MeSH) and free-text terms. First, we retrieved MeSH terms and their synonyms related to non-pharmacological interventions from the NCBI MeSH database, selecting representative terms based on semantic coverage. The final search strategy was determined after multiple rounds of pilot searching and refinement (see Supplementary material). To minimize omissions, we also manually searched the reference lists of relevant reviews. All retrieved records were imported into EndNote software and deduplicated using both automated and manual methods.

### 2.4 Data selection and extraction

Two reviewers (ZBF and BYQ) independently screened articles based on eligibility criteria through title, abstract, and full-text stages, and independently extracted data. Extracted items included: study characteristics (first author, publication year, and country), participant characteristics (sample size, sex, age), interventions (type, duration), and outcome measures (mean change from baseline and standard deviation). Data presented graphically were extracted using GetData software. When studies reported only baseline and post-intervention means and standard deviations without change-from-baseline standard deviations, these were estimated using the formula recommended by Follmann et al. (1992). Missing data or unavailable full texts were addressed by contacting original authors. Disagreements in screening or extraction were resolved through discussion or by a third reviewer (XLW) when necessary.

### 2.5 Risk of bias assessment

Two reviewers (LXL and ZQ) independently assessed the risk of bias of included randomized controlled trials using the Cochrane Risk of Bias 2.0 (RoB2) tool. This tool evaluates bias risk across five domains: (1) randomization process; (2) deviations from intended interventions; (3) missing outcome data; (4) outcome measurement; and (5) selective reporting. Each domain was rated as “low risk,” “some concerns,” or “high risk.” An overall risk of bias judgment was made for each study based on the domain ratings. Disagreements were resolved through discussion or consultation with a third reviewer (XLW) when necessary. Risk of bias results were presented as a risk of bias graph and bar chart.

### 2.6 Confidence in network meta-analysis

Based on risk of bias assessments, we used the CINeMA framework to rate the certainty of evidence for primary outcomes (Nikolakopoulou et al., 2020). CINeMA evaluates confidence in each comparison across six domains: (1) within-study bias; (2) reporting bias; (3) indirectness; (4) imprecision; (5) heterogeneity; and (6) incoherence. Each domain was judged as “no concerns,” “some concerns,” or “major concerns.” Judgments for imprecision, heterogeneity, and incoherence were made relative to minimally important differences of 1.3 points for the MMSE and 1.2 points for the MoCA. The overall certainty of evidence for each network estimate was rated as “high,” “moderate,” “low,” or “very low” (Papakonstantinou et al., 2020). The rating process was conducted using the CINeMA web application (https://cinema.med.auth.gr/).

### 2.7 Data synthesis and statistical analysis

Network meta-analysis was conducted using a frequentist random-effects model to integrate both direct and indirect evidence and evaluate the relative effectiveness of interventions. Analyses were performed using the netmeta package in R software (version 4.4.3).

First, we constructed a network graph to visualize the connections between interventions and the number of studies for each comparison. Node size was proportional to the total sample size of each intervention, and edge width represented the number of studies in each direct comparison, reflecting the density of the evidence network.

The model assumed consistency, and the between-study variance was estimated using the DerSimonian–Laird estimator. Main results included relative effect estimates with 95% confidence intervals presented as forest plots, and league tables summarizing all pairwise comparisons. Additionally, we used surface under the cumulative ranking curve (SUCRA) values to determine the relative ranking of each intervention, with higher SUCRA values indicating better treatment efficacy.

To assess statistical heterogeneity, we examined the I^2^ statistic and τ^2^ values. To examine the consistency assumption, we performed node-splitting analysis to detect local inconsistency between direct and indirect comparisons, and applied the design-by-treatment test to assess global inconsistency.

## 3 Results

### 3.1 Study selection and characteristics

A total of 978 records were identified from seven databases: PubMed (*n* = 95), Cochrane Library (*n* = 388), Embase (*n* = 172), Web of Science (*n* = 118), CNKI (*n* = 43), VIP (*n* = 36), and Wanfang (*n* = 126). After removing 421 duplicate records, 557 articles remained for title and abstract screening. Two researchers independently reviewed the titles and abstracts, excluding 235 irrelevant articles. The remaining 322 full-text articles were assessed for eligibility, of which 290 were excluded for the following reasons: duplicate publications (*n* = 3), non-aMCI diagnosis (*n* = 62), same intervention variants (*n* = 2), pharmacological intervention (*n* = 115), deficient data (*n* = 48), unmatched target outcomes (*n* = 56), and single-study intervention (*n* = 4). Ultimately, 32 randomized controlled trials were included in the network meta-analysis, comprising eleven different interventions. Among them, 22 studies used MMSE as the outcome measure and 21 studies used MoCA. The detailed screening process is presented in Figure 1.

**Figure 1:**
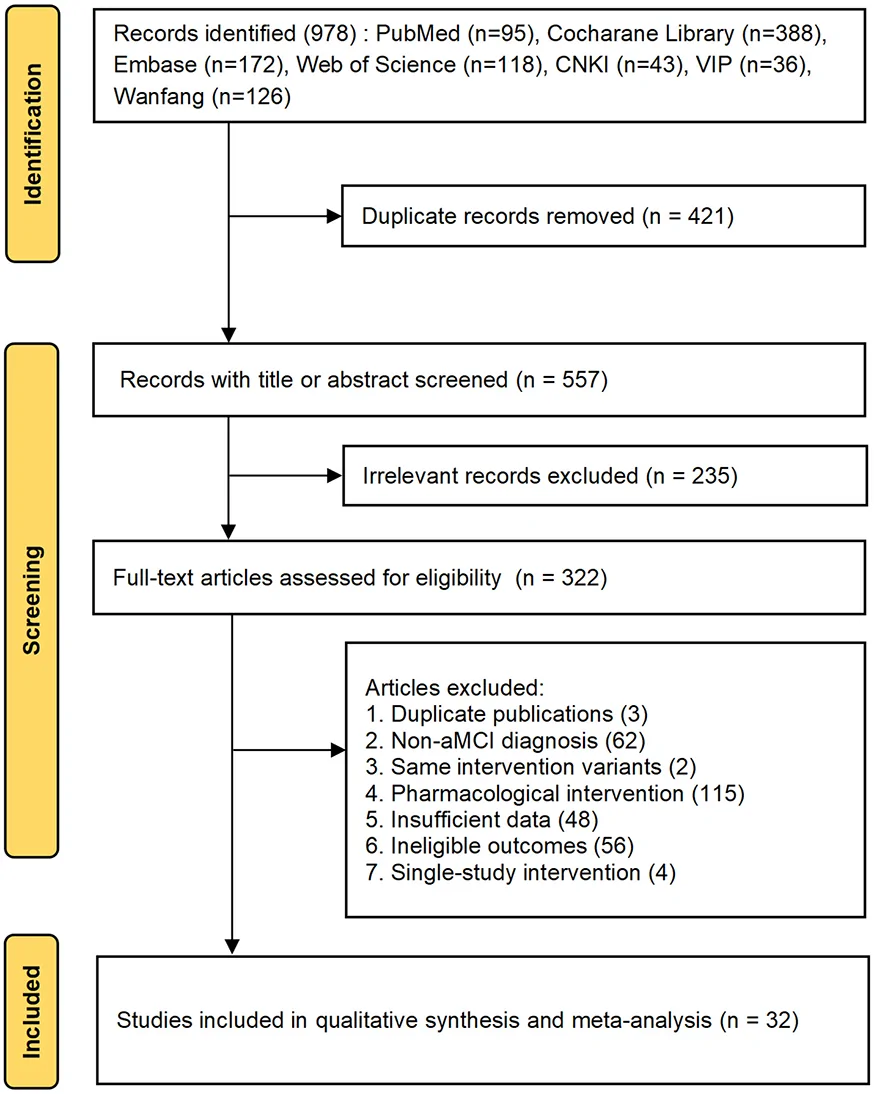
PRISMA 2020 flow diagram of study selection process.

A total of 2,142 participants were included in this study, who received eleven non-pharmacological interventions. The interventions were as follows: (1) moxibustion (Moxa, *n* = 202) (Liu et al., 2020; Li X. et al., 2023; Huang et al., 2024; Qin and Liu, 2023; Xiao et al., 2023; Wang et al., 2021; Zhao L. et al., 2025), (2) electroacupuncture (EA, *n* = 146) (Wen et al., 2020; Xu and Peng, 2017; Zhao et al., 2012), (3) manual acupuncture (MA, *n* = 170) (Li et al., 2020; Tan et al., 2017; Li Y. et al., 2023; Xu et al., 2024; Zhang, 2021; Zhang et al., 2025, 2026), (4) manual acupuncture plus moxibustion (MA_Moxa, *n* = 90) (Qin and Liu, 2023; Xiao et al., 2023; Zhu et al., 2015), (5) Tai Chi (TC, *n* = 185) (Lam et al., 2011; Bao and Liu, 2019; Yang et al., 2021), (6) cognitive behavioral therapy (CBT, *n* = 191) (Förster et al., 2011; Greenaway et al., 2013; Jeong et al., 2016; Nousia et al., 2023; Park et al., 2019; Feng et al., 2017; Savulich et al., 2017), (7) transcranial magnetic stimulation (TMS, *n* = 117) (Lv et al., 2024; Long et al., 2018; Wen et al., 2018; Chen et al., 2025; Wen et al., 2020), (8) active control (AC, *n* = 345), (9) placebo control (PC, *n* = 274), (10) treatment as usual (TAU, *n* = 265), and (11) no intervention (NI, *n* = 157). These studies were broadly categorized into four groups: traditional Chinese medicine (TCM) group, CBT group, TMS group, and control group.

The included studies were conducted across six countries, with the majority performed in China (*n* = 26, 81.3%), followed by Korea (*n* = 2, 6.3%), and one study each from Germany, the USA, Greece, and the UK (*n* = 1, 3.1% for each). The geographical distribution indicates a predominant focus on East Asian populations, particularly Chinese participants. Among the 32 included studies, 16 were reported in English and the remaining 16 were published in Chinese. The characteristics and extracted outcome data of each included study are summarized in Table 1.

**Table 1:** Characteristics and extracted outcome data of the 32 included randomized controlled trials.

Study (Year)	Intervention	*n*	Female *n*/*N* (%)^a^	Age^b^ (years)	Duration (weeks)	MMSE, mean ±SD		MoCA, mean ±SD	
						Baseline	Δ^c^	Baseline	Δ^c^
Förster et al. (2011)	CBT	9	NR	NR	24	28.10 ± 1.60	0.20 ± 1.44	—	—
	AC	12		NR		26.80 ± 1.50	-0.80 ± 1.41	—	—
Greenaway et al. (2013)	CBT	20	24/40 (60.0)	72.30 ± 7.90	24	26.40 ± 2.20	-0.20 ± 1.40	—	—
	NI	20		72.70 ± 6.90		27.20 ± 2.40	-0.40 ± 2.20	—	—
Jeong et al. (2016)	CBT	71	141/224 (62.9)	70.80 ± 6.90	12	25.90 ± 2.50	0.60 ± 2.10	—	—
	AC	77		68.50 ± 8.50		25.90 ± 2.40	0.40 ± 1.80	—	—
	NI	76		71.60 ± 6.50		25.30 ± 2.50	0.30 ± 1.80	—	—
Lam et al. (2011)	TC	135	NR	>65	8	24.80 ± 3.10	1.00 ± 3.10	—	—
	AC	194		>65		24.20 ± 2.90	0.90 ± 3.30	—	—
Li et al. (2020)	MA	14	NR	NR	24	25.00 ± 3.10	0.80 ± 2.60	—	—
	NI	14		NR		25.40 ± 3.70	-1.40 ± 1.70	—	—
Liu et al. (2020)	Moxa	28	31/54 (57.4)	60.00 ± 4.00	8	24.54 ± 0.84	3.32 ± 1.18	22.43 ± 3.04	3.79 ± 3.61
	PC	26		62.00 ± 5.00		24.50 ± 0.86	0.77 ± 2.05	23.15 ± 3.17	0.54 ± 3.69
Lv et al. (2024)	TMS	39	30/60 (50.0)	66.74 ± 1.08	3	—	—	22.46 ± 3.61	1.93 ± 3.34
	PC	21		64.95 ± 8.72		—	—	23.43 ± 4.98	-0.76 ± 4.60
Nousia et al. (2023)	CBT	15	14/30 (46.7)	75.73 ± 4.48	15	—	—	22.73 ± 1.44	0.94 ± 1.52
	TAU	15		76.67 ± 3.81		—	—	22.07 ± 1.71	0.87 ± 1.60
Park et al. (2019)	CBT	25	NR	70.70 ± 7.50	24	25.50 ± 2.50	0.20 ± 2.40	—	—
	PC	24		69.70 ± 8.40		25.90 ± 2.80	–0.10 ± 3.10	—	—
Tan et al. (2017)	MA	16	16/32 (50.0)	65.88 ± 4.66	4	22.25 ± 1.84	2.88 ± 1.45	22.69 ± 1.58	2.00 ± 1.10
	PC	16		64.56 ± 5.25		23.31 ± 2.70	0.06 ± 0.86	23.63 ± 1.20	–0.19 ± 0.83
Li X. et al. (2023)	Moxa	16	NR	NR	8	24.60 ± 0.90	2.70 ± 2.30	22.80 ± 2.70	3.30 ± 2.20
	PC	16		NR		24.50 ± 0.80	0.90 ± 1.30	23.30 ± 3.00	0.60 ± 0.70
Bao and Liu (2019)	TC	31	33/62 (53.2)	68.22 ± 9.84	24	17.31 ± 5.26	7.81 ± 4.80	—	—
	AC	31		65.62 ± 9.34		17.55 ± 5.40	0.36 ± 5.54	—	—
Feng et al. (2017)	CBT	30	33/60 (55.0)	81.40 ± 3.60	12	21.61 ± 3.07	2.36 ± 2.84	—	—
	NI	30		81.30 ± 3.60		21.79 ± 3.11	0.15 ± 3.19	—	—
Huang et al. (2024)	Moxa	18	19/34 (55.9)	59.10 ± 4.10	8	24.56 ± 0.86	3.05 ± 1.60	23.00 ± 2.63	3.11 ± 2.60
	PC	16		61.30 ± 5.20		24.50 ± 0.82	0.94 ± 1.20	23.31 ± 3.00	0.57 ± 2.80
Li Y. et al. (2023)	MA	30	39/60 (65.0)	62.80 ± 7.70	4	23.84 ± 2.11	3.59 ± 2.17	19.69 ± 1.91	5.03 ± 2.83
	TAU	30		64.50 ± 7.40		22.99 ± 2.57	2.93 ± 3.22	20.16 ± 2.57	3.06 ± 3.99
Long et al. (2018)	TMS	15	16/30 (53.3)	68.27 ± 9.85	1.5	—	—	26.47 ± 0.64	0.53 ± 0.83
	PC	15		65.63 ± 9.36		—	—	26.50 ± 0.88	0.10 ± 0.91
Qin and Liu (2023)	MA_Moxa	30	38/60 (63.3)	65.20 ± 4.77	8	—	—	20.10 ± 2.56	4.97 ± 2.48
	Moxa	30		63.63 ± 5.56		—	—	20.07 ± 1.91	3.06 ± 1.95
Wen et al. (2018)	TMS	23	21/45 (46.7)	64.17 ± 5.21	4	—	—	22.30 ± 1.36	2.79 ± 1.24
	PC	22		65.91 ± 4.93		—	—	21.82 ± 1.62	–0.09 ± 1.50
Wen et al. (2020)	TMS	23	27/45 (60.0)	65.87 ± 4.82	4	—	—	22.35 ± 1.15	1.87 ± 1.59
	EA	22		64.59 ± 5.78		—	—	21.91 ± 1.41	1.83 ± 1.67
Xiao et al. (2023)	MA_Moxa	30	60/60 (100)	50–75	8	24.33 ± 1.24	3.50 ± 1.30	22.70 ± 1.51	4.03 ± 1.82
	Moxa	30		50–75		24.47 ± 1.11	1.60 ± 1.90	22.57 ± 1.33	2.10 ± 2.05
Xu et al. (2024)	MA	40	50/80 (62.5)	57.04 ± 3.18	8	—	—	20.05 ± 1.88	5.00 ± 2.17
	PC	40		66.24 ± 3.55		—	—	20.08 ± 2.54	3.03 ± 2.30
Xu and Peng (2017)	EA	30	28/60 (46.7)	62.12 ± 8.01	8	24.49 ± 0.62	3.23 ± 1.50	24.53 ± 0.60	3.17 ± 1.10
	TAU	30		61.20 ± 7.63		24.49 ± 0.62	2.46 ± 1.25	24.05 ± 0.11	2.95 ± 1.01
Zhang (2021)	MA	17	20/35 (57.1)	64.59 ± 7.74	12	23.71 ± 1.90	3.11 ± 1.83	21.06 ± 2.70	4.18 ± 2.40
	PC	18		61.83 ± 7.37		24.44 ± 2.55	1.12 ± 2.30	21.00 ± 1.85	2.22 ± 1.71
Zhu et al. (2015)	MA_Moxa	30	27/60 (45.0)	62.13 ± 7.99	8	24.50 ± 0.63	3.23 ± 1.55	24.37 ± 1.13	3.20 ± 1.45
	TAU	30		60.90 ± 7.52		24.50 ± 0.63	2.47 ± 1.31	24.07 ± 1.11	2.80 ± 1.19
Zhao et al. (2012)	EA	94	97/187 (51.9)	69.00 ± 7.00	8	25.14 ± 0.92	3.29 ± 1.23	—	—
	TAU	93		67.00 ± 6.00		25.20 ± 0.95	2.09 ± 1.22	—	—
Wang et al. (2021)	Moxa	49	66/95 (69.5)	72.30 ± 8.60	8	—	—	22.33 ± 1.07	3.41 ± 1.01
	TAU	46		70.30 ± 9.40		—	—	22.82 ± 1.04	2.49 ± 1.17
Zhang et al. (2025)	MA	29	37/53 (69.8)	59.03 ± 6.84	8	26.31 ± 1.49	2.66 ± 1.31	22.69 ± 2.14	4.41 ± 1.96
	PC	24		61.71 ± 7.50		26.67 ± 1.58	1.29 ± 1.63	22.79 ± 2.55	1.42 ± 2.43
Yang et al. (2021)	TC	19	26/36 (72.2)	83.16 ± 5.70	12	25.10 ± 1.40	1.40 ± 1.64	—	—
	NI	17		82.06 ± 5.50		25.40 ± 1.30	–0.20 ± 2.43	—	—
Chen et al. (2025)	TMS	17	19/27 (70.4)	67.00 ± 7.00	4	—	—	24.00 ± 5.66	1.00 ± 5.05
	PC	10		68.00 ± 8.75		—	—	24.50 ± 3.66	-0.50 ± 4.02
Savulich et al. (2017)	CBT	21	17/42 (40.5)	75.20 ± 7.40	4	26.60 ± 2.90	0.80 ± 2.50	—	—
	TAU	21		76.90 ± 9.30		26.80 ± 2.20	-0.70 ± 2.30	—	—
Zhang et al. (2026)	MA	24	33/50 (66.0)	61.19 ± 7.19	8	26.31 ± 1.52	2.69 ± 1.33	22.92 ± 1.98	4.16 ± 1.89
	PC	26		61.63 ± 7.60		26.63 ± 1.58	1.21 ± 1.65	22.54 ± 2.62	1.59 ± 2.46
Zhao et al. ((2025)	Moxa	31	33/62 (53.2)	63.03 ± 5.46	12	—	—	23.32 ± 2.18	2.55 ± 1.91
	AC	31		62.61 ± 5.43		—	—	22.55 ± 2.22	0.64 ± 2.75

Values are mean ± SD. NR, not reported; dashes (—) indicate that the instrument was not used in the analyses for that study. ^a^Sex distribution at the study level. ^b^Age at baseline. ^c^ Δ = post-intervention minus baseline, with positive values indicating improvement. AC, active control; CBT, cognitive behavioral therapy; EA, electroacupuncture; MA, manual acupuncture; MA_Moxa, manual acupuncture plus moxibustion; Moxa, moxibustion; NI, no intervention; PC, placebo control; TAU, treatment as usual; TC, Tai Chi; TMS, transcranial magnetic stimulation.

### 3.2 Risk of bias assessment

The risk of bias for the included studies is presented in Supplementary Figures S1, S2. Regarding the randomization process, 19 studies (59.4%) were rated as low risk, employing appropriate randomization methods such as computer-generated random numbers, block randomization, or stratified randomization, while 13 studies (40.6%) raised some concerns due to insufficient reporting of specific allocation methods or lack of information about allocation concealment. For deviations from intended interventions, 30 studies (93.8%) were rated as low risk; due to the inherent nature of non-pharmacological interventions, blinding of participants and personnel was challenging, though most studies maintained low risk of bias in this domain, with only 2 studies (6.2%) raising some concerns. Concerning missing outcome data, 30 studies (93.8%) were rated as low risk, reporting complete data or employing appropriate methods to handle missing data, whereas 2 studies (6.2%) raised some concerns. Most studies reported participant dropout, with the majority demonstrating acceptable dropout rates. For measurement of the outcome, 27 studies (84.4%) were rated as low risk, using validated and reliable assessment tools for cognitive outcomes, while 5 studies (15.6%) raised some concerns. Regarding selection of the reported result, 24 studies (75.0%) were rated as low risk, with no evidence of selective reporting or deviation from pre-specified analysis plans, whereas 8 studies (25.0%) raised some concerns. Overall, 15 studies (46.9%) were rated as low risk and 17 studies (53.1%) raised some concerns; no study was rated as high risk. The overall methodological quality of the included studies was deemed acceptable.

### 3.3 Pairwise meta-analysis results

Pairwise meta-analyses were conducted using a random-effects model for each intervention compared with TAU for MMSE and MoCA outcomes. The results are presented in Supplementary Figures S3, S4. For MMSE outcome, among the 10 interventions compared with TAU, only MA_Moxa demonstrated a statistically significant improvement (MD = 1.65, 95% CI: 0.01 to 3.29). No significant differences were observed for the remaining interventions. For MoCA outcome, among the 8 interventions compared with TAU, only MA_Moxa demonstrated a statistically significant improvement (MD = 1.68, 95% CI: 0.30 to 3.06). Overall, MA_Moxa demonstrated consistent statistically significant effects on both MMSE and MoCA outcomes compared with TAU.

### 3.4 Network meta-analysis results

Network meta-analyses were performed separately for MMSE and MoCA outcomes (network plots shown in Figure 2). For MMSE, MA_Moxa achieved the highest SUCRA probability (87.9%), followed by TC (76.7%) and EA (71.7%). For MoCA, MA_Moxa again demonstrated the highest probability of being the best treatment (SUCRA = 95.3%), followed by MA (71.5%) and Moxa (65.9%); the full ranking distributions are presented in the rankogram (Figure 3). Compared with TAU, MA_Moxa showed statistically significant improvement in MMSE scores (MD = 1.65, 95% CI 0.01 to 3.29). For MoCA, MA_Moxa was significantly more effective than TAU (MD = 1.68, 95% CI 0.30 to 3.06), whereas PC was significantly less effective than TAU (MD = −1.82, 95% CI −3.22 to −0.42). No statistically significant differences were observed between TAU and the remaining interventions; complete pairwise comparisons are provided in the league table (Figure 4). TCM interventions consistently achieved higher rankings than non-TCM controls across both cognitive outcomes.

**Figure 2:**
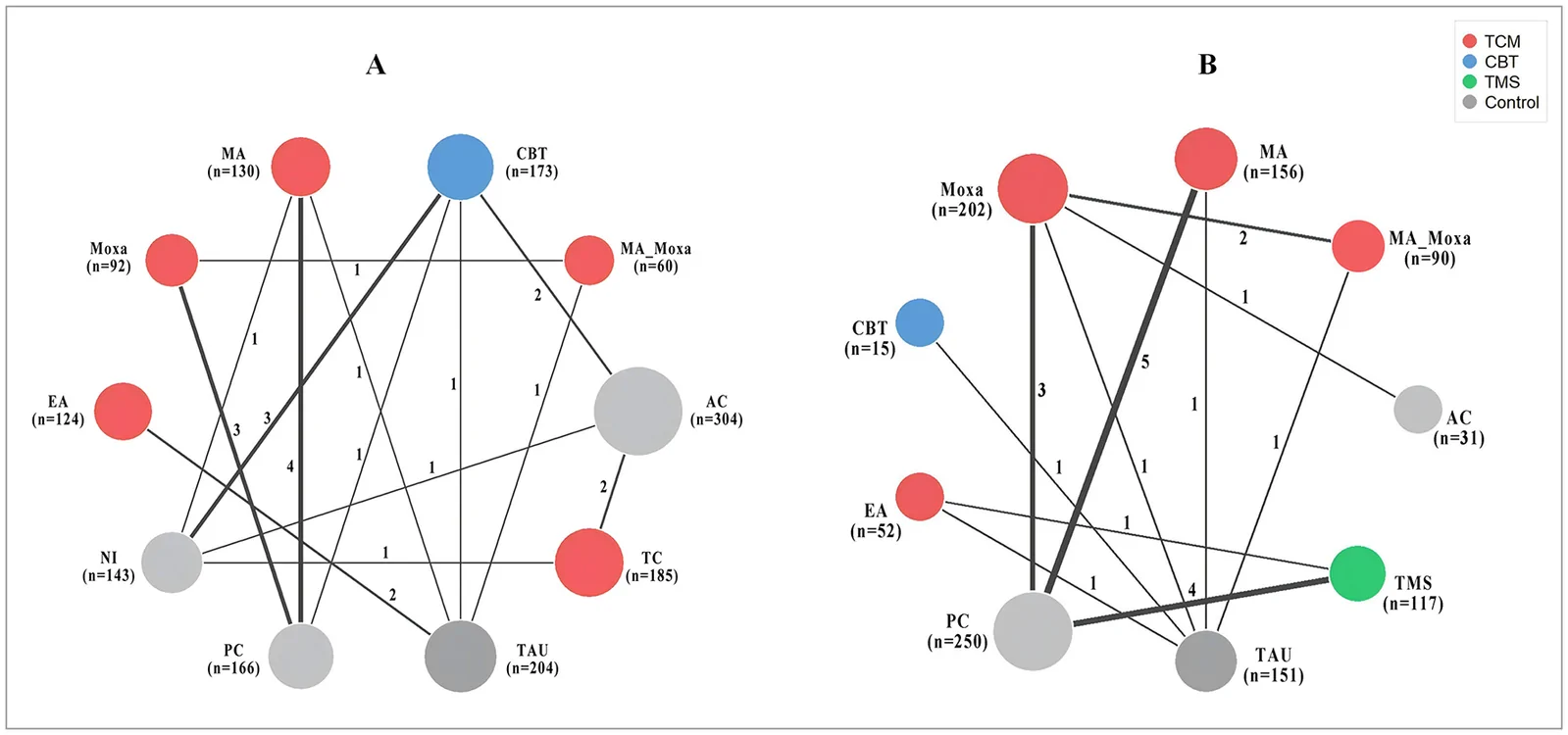
Network plots of eligible comparisons for **(A)** MMSE and **(B)** MoCA outcomes. Node size reflects the number of participants receiving each intervention, and edge width reflects the number of studies informing each direct comparison.

**Figure 3:**
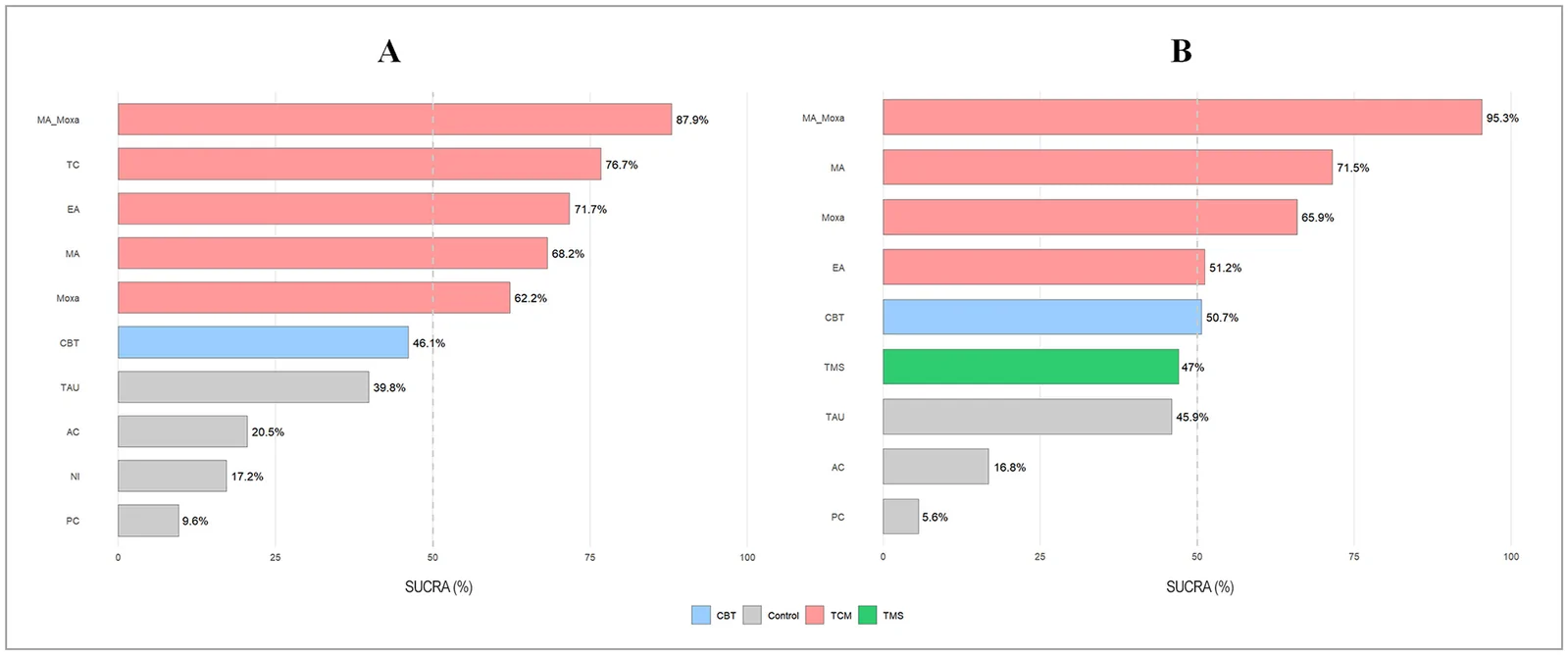
Surface under the cumulative ranking curve (SUCRA) probabilities for **(A)** MMSE and **(B)** MoCA outcomes. Higher SUCRA values indicate a higher probability of being the best treatment.

**Figure 4:**
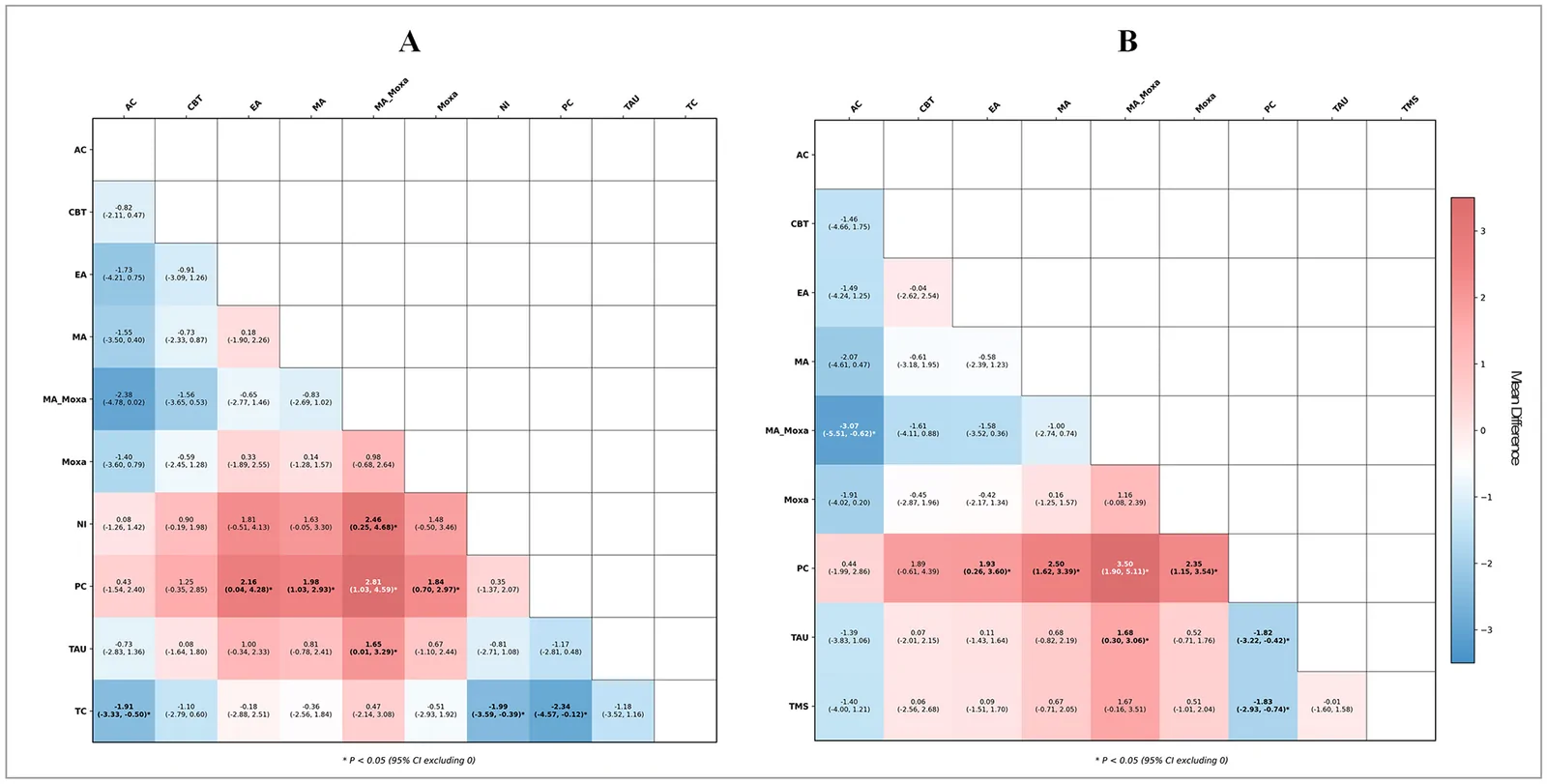
League tables presenting pairwise comparisons for **(A)** MMSE and **(B)** MoCA outcomes. Each cell shows the mean difference (95% confidence interval) for the row-defining intervention compared with the column-defining intervention. Statistically significant results (*P* < 0.05) are marked with an asterisk (*).

### 3.5 Heterogeneity and consistency assessment

Heterogeneity and consistency were assessed for both MMSE and MoCA outcomes (Table 2). For MMSE, substantial heterogeneity was observed (τ^2^ = 0.85, I^2^ = 77.6%, *Q* = 62.51, *df* = 14, *p* < 0.001), with no statistically significant inconsistency detected by the SIDE splitting method. For MoCA, considerable heterogeneity was also present (τ^2^ = 0.80, I^2^ = 73.8%, *Q* = 49.55, *df* = 13, *p* < 0.001). The SIDE splitting method revealed statistically significant local inconsistency for two comparisons involving MA_Moxa. For MA_Moxa vs. Moxa, the direct and indirect estimates pointed in opposite directions (direct MD = 1.92 vs. indirect MD = −0.88; *p* = 0.047); for MA_Moxa vs. TAU, both estimates favored MA_Moxa but differed markedly in magnitude (direct MD = 0.40 vs. indirect MD = 3.20; *p* = 0.047). Because MA_Moxa was informed by only three small trials, this inconsistency materially reduces confidence in the corresponding network estimates. The design-by-treatment test with random-effects weighting was non-significant for both outcomes (MMSE: *Q* = 4.99, *df* = 6, *p* = 0.545; MoCA: *Q* = 4.71, *df* = 3, *p* = 0.194), indicating no global inconsistency. A sensitivity analysis excluding the MA_Moxa node likewise found no global inconsistency for either outcome (MMSE: *Q* = 2.46, *df* = 5, *p* = 0.783; MoCA: *Q* = 0.99, *df* = 2, *p* = 0.610), and the hierarchy of the remaining interventions was preserved (see Section 3.6.1), indicating that the incoherence is localized to MA_Moxa.

**Table 2:** Heterogeneity and consistency assessment.

Outcome	Heterogeneity						SIDE splitting inconsistent comparisons	Design-by-treatment test^a^		
MMSE	0.85	62.51	14	<0.001	77.6%	High	0	4.99	6	0.545
MoCA	0.80	49.55	13	<0.001	73.8%	Substantial	2	4.71	3	0.194

τ^2^, between-study variance; *Q*, Cochran's Q statistic; df, degrees of freedom; I^2^, percentage of total variation across studies due to heterogeneity. SIDE, separating indirect from direct evidence. ^a^Design-by-Treatment test with random-effects weighting.

### 3.6 Robustness assessment and heterogeneity exploration

#### 3.6.1 Sensitivity analysis

For MoCA (*n* = 21), three studies were identified as influential: Nousia et al. (2023) evaluating CBT vs. TAU in Greece (*n* = 15), Tan et al. (2017) evaluating manual acupuncture vs. placebo control in China (*n* = 16), and Long et al. (2018) evaluating TMS vs. placebo control in China (*n* = 15, intervention duration 1.5 weeks). Exclusion of these studies resulted in I^2^ reductions of 19.2%, 10.5%, and 16.0%, respectively. For MMSE (*n* = 22), exclusion of Tan et al. (2017) yielded the most substantial reduction in heterogeneity (I^2^ decreased from 77.6% to 62.9%). These studies were characterized by small sample sizes (*n* = 15–16) and short intervention durations (1.5–4 weeks). Despite the influence of these individual studies, the overall treatment rankings and effect directions remained consistent across all sensitivity scenarios, indicating robustness of the primary findings. Furthermore, given the local inconsistency involving the MA_Moxa node (Section 3.5), we repeated both network meta-analyses after excluding the three trials informing this node. No global inconsistency was detected after exclusion (MMSE: *Q* = 2.46, *df* = 5, *p* = 0.783; MoCA: *Q* = 0.99, *df* = 2, *p* = 0.610). The hierarchy of the remaining interventions was preserved: moxibustion, Tai Chi, and manual acupuncture remained the top-ranked interventions for MMSE (SUCRA 85.7%, 80.2%, and 78.1%), and moxibustion and manual acupuncture for MoCA (SUCRA 88.5% and 83.6%), with moxibustion showing a statistically significant improvement over TAU for MoCA (MD = 1.24, 95% CI 0.05 to 2.44). These findings indicate that the inconsistency is localized to the MA_Moxa node and that the relative ordering of the remaining interventions is robust; the top ranking of MA_Moxa should therefore be regarded as tentative. Notably, Traditional Chinese Medicine–based interventions (moxibustion, manual acupuncture, Tai Chi, and electroacupuncture) remained clustered at the top of the hierarchy for both outcomes regardless of whether the MA_Moxa node was included. Detailed results are presented in Supplementary Tables S1, S2.

#### 3.6.2 Publication bias

Publication bias was assessed using Egger's linear regression test, Begg's rank correlation test, and the Trim and Fill method. Significant asymmetry was detected for MMSE (Egger: intercept = 2.79, *p* < 0.001; Begg: τ = 0.38, *p* = 0.010) and MoCA (Egger: intercept = 2.24, *p* = 0.002; Begg: τ = 0.28, *p* = 0.080). Trim and Fill analysis imputed 6 studies for MMSE and 7 studies for MoCA, with adjusted pooled estimates of MD = 0.92 (95% CI 0.37 to 1.46) and MD = 1.04 (95% CI 0.50 to 1.58), respectively. Although adjusted estimates remained directionally consistent with the primary analysis, the magnitude of effects was attenuated by approximately 31% for MMSE and 36% for MoCA, suggesting potential overestimation in the primary analysis. Detailed results are presented in Supplementary Table S3.

#### 3.6.3 Meta-regression

Meta-regression was conducted to explore sources of heterogeneity, with sample size, female proportion, mean age, baseline cognitive scores, and intervention duration as covariates. Analyses were performed on comparison-level mean differences in change-from-baseline scores using random-effects models (restricted maximum likelihood), with each covariate entered separately. For MMSE (22 studies contributing 23 comparisons), larger sample sizes were associated with significantly smaller treatment effects (β = −0.0061 per participant, *p* = 0.004, R^2^ = 46.9%), consistent with small-study effects, and higher baseline cognitive scores were associated with smaller improvements (β = −0.4710 per point, *p* < 0.001, R^2^ = 41.5%). Female proportion, mean age, and intervention duration showed no significant associations (all *p*>0.05). For MoCA (*n* = 21), no moderator was statistically significant in both the primary and sensitivity analyses: the nominally significant negative association with mean age (β = −0.1209, *p* = 0.042, *R*^2^ = 13.5%; uncorrected for multiplicity across the five covariates tested) lost significance after excluding two studies with estimated age values (*p* = 0.056). Neither baseline severity nor sample size significantly moderated MoCA effect estimates; both associations were nevertheless directionally consistent with those observed for MMSE, and the narrower covariate ranges across MoCA trials (baseline 19.9–26.5; sample size 27–95) may have limited the statistical power to detect such moderation. These findings indicate that heterogeneity in MMSE outcomes was mainly attributable to small-study effects and differences in baseline severity, whereas heterogeneity in MoCA outcomes remains largely unexplained by the study-level characteristics examined. Detailed results are presented in Table 3.

**Table 3:** Results of meta-regression analyses.

Covariate	Coefficient (SE)	*p*-value	*R*^2^ (%)
MMSE (*n* = 22)			
Sample size	−0.0061 (0.0021)	**0.004**	46.9
Female proportion	−0.0077 (0.0173)	0.657	0
Intervention duration	0.0038 (0.0354)	0.914	0
Age	−0.0207 (0.0359)	0.564	0
Baseline score	−0.4710 (0.1160)	**<0.001**	41.5
MoCA (*n* = 21)			
Sample size	−0.0032 (0.0138)	0.815	0
Female proportion	0.0187 (0.0191)	0.328	0.5
Intervention duration	−0.0203 (0.0772)	0.793	0
Age	−0.1209 (0.0594)	**0.042**	13.5
Baseline score	−0.2495 (0.1459)	0.087	16.2

SE, standard error; *R*^2^, proportion of between-study variance explained. Analyses were performed on comparison-level mean differences in change-from-baseline scores (random-effects models, restricted maximum likelihood), with each covariate entered separately; for MMSE, 22 studies contributed 23 comparisons (one three-arm trial contributed two comparisons). **Bold** *p*-values indicate statistical significance (*p* < 0.05).

### 3.7 Certainty of evidence

The certainty of evidence for each network estimate was rated using the CINeMA framework. For MMSE (45 comparisons), confidence was rated as moderate for 38 comparisons and very low for 7 comparisons; for MoCA (36 comparisons), confidence was moderate for 27 and very low for 9. Very-low ratings were driven mainly by major concerns in heterogeneity and, for comparisons involving MA_Moxa, by major concerns in incoherence, reflecting the local inconsistency identified by node-splitting (Section 3.5). Full domain-level judgments are presented in Supplementary Figures S5, S6.

## 4 Discussion

This network meta-analysis of 32 RCTs provides comprehensive evidence on non-pharmacological interventions for aMCI. Traditional Chinese Medicine, particularly the combination of manual acupuncture and moxibustion (MA_Moxa), demonstrated the highest probability of being the best treatment (SUCRA 87.9% for MMSE, 95.3% for MoCA), although this ranking should be regarded as tentative given the local inconsistency discussed below. Overall, effect sizes across interventions were modest, likely reflecting the inherent treatment resistance of aMCI, a condition characterized by progressive neurodegeneration that is difficult to reverse. This finding aligns with the view that MCI represents a transitional stage in which cognitive decline, while potentially modifiable, is challenging to fully arrest through non-pharmacological means alone.

The meta-regression identified sample size and baseline cognitive score as significant moderators of MMSE outcomes. Treatment effects were larger in smaller trials (β = −0.0061 per participant, *p* = 0.004) and in studies with lower baseline scores (β = −0.4710 per point, *p* < 0.001). The first association is consistent with the small-study effects detected in our publication-bias analyses, in which Trim and Fill adjustment attenuated the pooled effects by approximately one third. The second likely reflects greater room for improvement, or regression to the mean, among more severely impaired participants. No moderator for MoCA remained significant in sensitivity analyzes.

The modest absolute effect sizes deserve careful interpretation. The improvements of 1.65 and 1.68 points for MA_Moxa over TAU on the MMSE and MoCA fall below thresholds considered clinically meaningful at the individual level. The minimal clinically important difference in MCI is approximately 1–3 MMSE points (Andrews et al., 2019; Muir et al., 2024), and the minimal detectable change (MDC_95_) has been estimated at 3 points for the MMSE and 4 points for the MoCA (Feeney et al., 2016). Year-to-year within-person variation on the MoCA commonly reaches 2–3 points even in cognitively unimpaired older adults, and only changes of at least 4 points exceed the normal range of variation (Malek-Ahmadi et al., 2018). Our estimates should thus be read as population-level average effects rather than individually perceptible benefits, and they are likely inflated to some extent by the small-study effects noted above. Even so, for a highly prevalent condition without approved disease-modifying pharmacotherapy, modest average improvements may remain meaningful at the population level, particularly given the favorable safety and cost profiles of these interventions.

We also note the well-documented phenomenon of reversion from MCI to normal cognition. Approximately 24% of individuals with MCI have been estimated to revert to normal cognition, with rates differing between clinic-based (14%) and community-based (31%) samples (Malek-Ahmadi, 2016); a more recent meta-analysis reported a pooled reversion rate of 31% (Zhao Y. et al., 2025). Because none of the included trials reported reversion as an outcome, part of the observed improvement, particularly in control groups, may reflect natural fluctuation rather than intervention effects. This reinforces the need for cautious, clinically anchored interpretation of the pooled effect sizes.

Regarding the intervention combinations, this analysis included only one combination therapy (MA_Moxa) due to the limited availability of studies evaluating other combined approaches. Several considerations informed this decision. Clinically, combination therapies are common in practice, but their effects are not simply additive: synergistic, antagonistic, or modulatory interactions may occur. Methodologically, including multiple combination therapies with sparse data would compromise network connectivity and estimation precision, as the NMA framework requires adequate connections between nodes. Furthermore, disaggregating combined interventions into constituent components would violate the “combination as a whole” principle, potentially misrepresenting the true therapeutic interaction. MA_Moxa was therefore retained, as it had sufficient data and represents a clinically relevant combination commonly used in traditional Chinese medicine practice.

The node-splitting results for MA_Moxa deserve particular attention. Direct and indirect evidence disagreed in direction for MA_Moxa vs. Moxa and MA_Moxa vs. TAU, and the MA_Moxa node rested on only three small trials with heterogeneous protocols and selected populations. Although the remainder of the network was robust to exclusion of this node, the top SUCRA ranking of MA_Moxa is tentative and should not be taken as evidence of superiority over single-modality interventions. Adequately powered head-to-head trials comparing combined acupuncture–moxibustion with each component alone are needed to resolve this question.

With respect to outcome measures, MMSE and MoCA were analyzed separately rather than combined using standardized mean differences. This decision was grounded in the distinct cognitive domains assessed by these instruments: MMSE predominantly evaluates memory and orientation with limited sensitivity to executive function, whereas MoCA was specifically designed to detect mild cognitive impairment, incorporating tasks that assess executive function, working memory, and visuospatial abilities, domains typically affected earlier in the MCI spectrum. Given the differential sensitivity and construct validity of these measures, their interchangeable use or statistical combination would be inappropriate. Indeed, our separate analyses revealed notable differences in intervention rankings between outcomes, underscoring the importance of domain-specific assessment in MCI research. Future studies should consider employing both instruments to capture the multidimensional nature of cognitive impairment.

Publication bias was assessed using Egger's linear regression test, Begg's rank correlation test, and the Trim and Fill method. Significant asymmetry was detected for MMSE (Egger: *p* < 0.001; Begg: *p* = 0.010) and MoCA (Egger: *p* = 0.002; Begg: *p* = 0.080). Trim and Fill analysis imputed 6 studies for MMSE and 7 studies for MoCA, with adjusted pooled estimates showing attenuated but directionally consistent effects (approximately 31% reduction for MMSE, 36% for MoCA). These findings indicate the presence of small-study effects, wherein smaller trials with favorable results may be preferentially published. However, the consistent direction of treatment effects after adjustment supports the conclusion that these interventions confer genuine, albeit modest, cognitive benefits.

Several limitations warrant consideration. First, significant heterogeneity was observed across studies (I^2^ = 77.6% for MMSE, 73.8% for MoCA), reflecting variability in intervention protocols and participant characteristics. Second, the network geometry revealed limited direct comparisons between certain intervention pairs, with some comparisons relying heavily on indirect evidence. Third, the local inconsistency involving the MA_Moxa node reduces confidence in its network estimates, and its top ranking should be interpreted as tentative. Fourth, the median follow-up duration of 12 weeks limits conclusions regarding the sustainability of cognitive benefits. Fifth, although we assessed publication bias using multiple methods, the presence of small-study effects and potential selective reporting cannot be entirely excluded.

Despite these limitations, our findings have implications for clinical practice. Given their favorable safety profiles, non-pharmacological interventions represent a valuable component of aMCI management; however, the modest effect sizes, likely inflated by small-study effects, support their use as adjunctive rather than stand-alone therapies. The hierarchical ranking provided by our analysis may assist clinicians in selecting interventions based on available resources and patient preferences. Future research should prioritize longer follow-up periods, adequately powered head-to-head comparisons between active interventions (including combined vs. single-modality regimens), and studies in treatment-resistant sub-populations.

## 5 Conclusions

This network meta-analysis suggests that non-pharmacological interventions are a valuable adjunctive option for aMCI management, with traditional Chinese medicine-based modalities consistently occupying the top ranks for both cognitive outcomes and combined manual acupuncture and moxibustion ranking highest. However, the effects on cognitive scores were modest, the top ranking of the combined intervention is tentative given the local inconsistency and the small number of informing trials, and meta-regression indicated that pooled effects were larger in smaller trials and in studies with lower baseline cognitive scores, underscoring the need for cautious interpretation. Methodological constraints regarding combination therapies and outcome measures reflect the complexity of NMA implementation in heterogeneous intervention landscapes. Future research should prioritize long-term efficacy trials and adequately powered head-to-head comparisons of combined vs. single-modality interventions.

## Data Availability

The original contributions presented in the study are included in the article/Supplementary material, further inquiries can be directed to the corresponding author.
