# Supplementary material for: Comparative efficacy of non-pharmacological interventions for amnestic mild cognitive impairment: a network meta-analysis
Source: Front Aging Neurosci. 2026 Oct 2;18:1878590. doi: 10.3389/fnagi.2026.1878590 (PMC13672645; doi:10.3389/fnagi.2026.1878590)
Supplement: Supplementary file 1 [file Data_Sheet_1.pdf]

Supplementary Information

SUPPLEMENTARY FIGURES

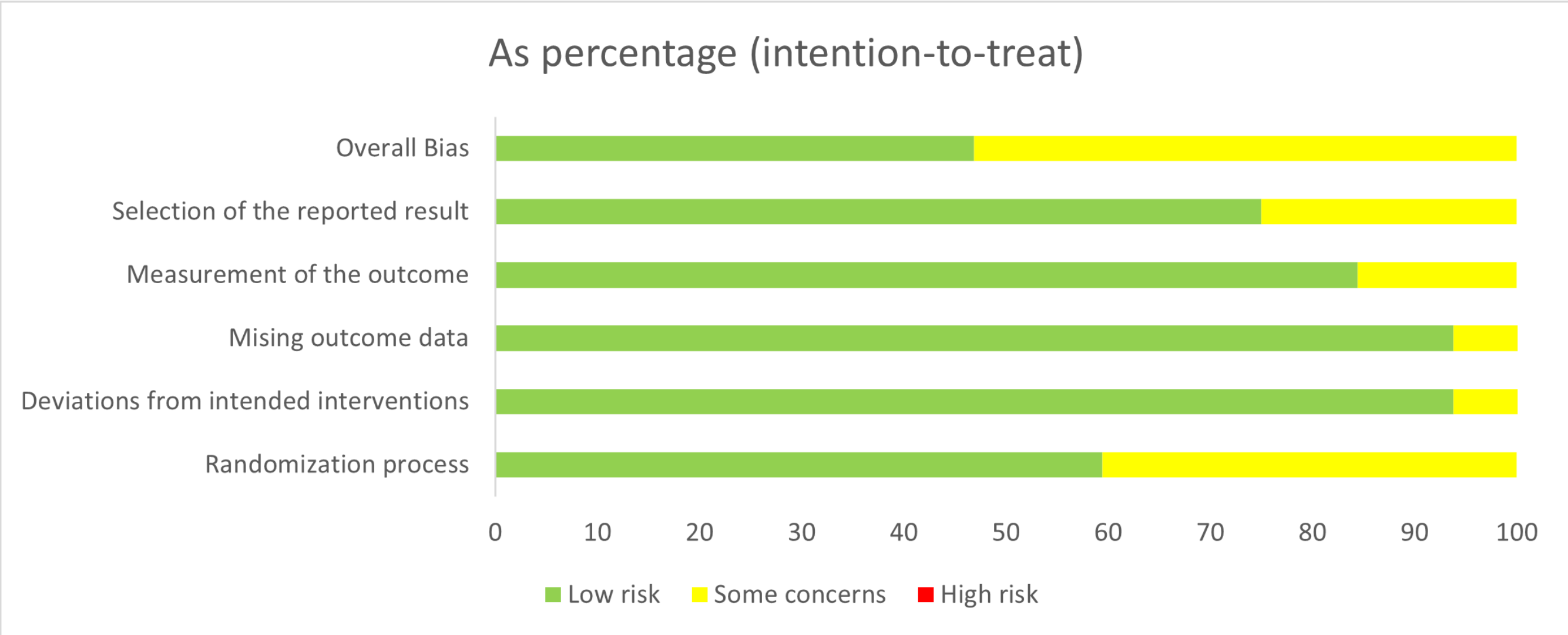

Figure S1. Risk of bias summary bar chart (RoB2).

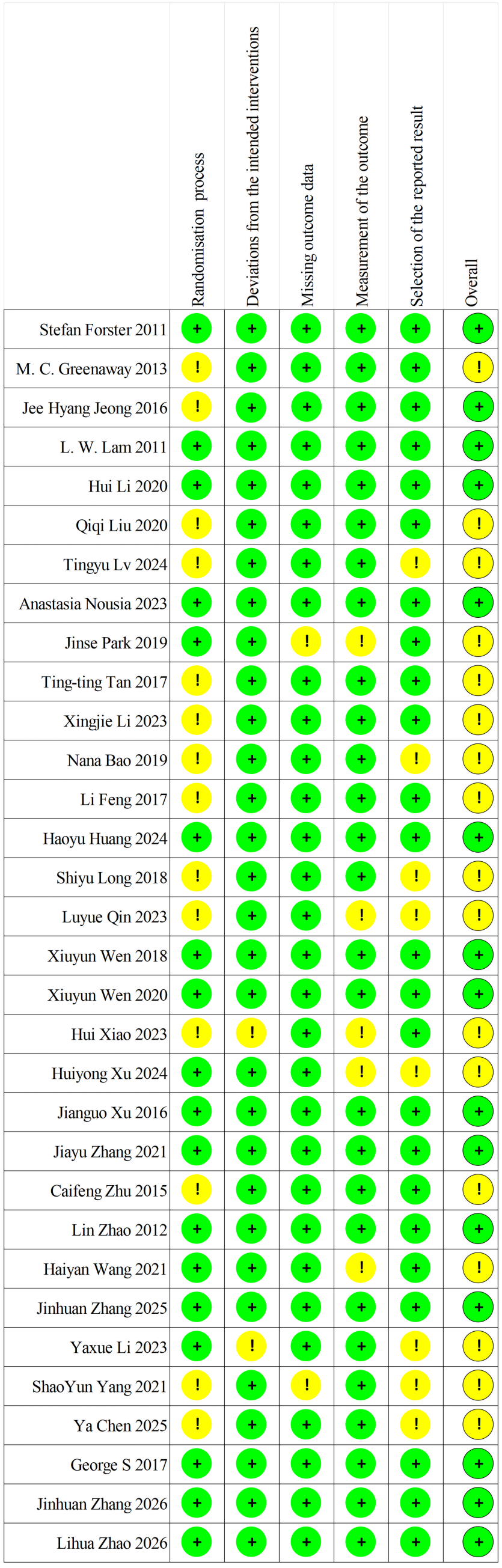

Figure S2. Risk of bias traffic light plot (RoB2).

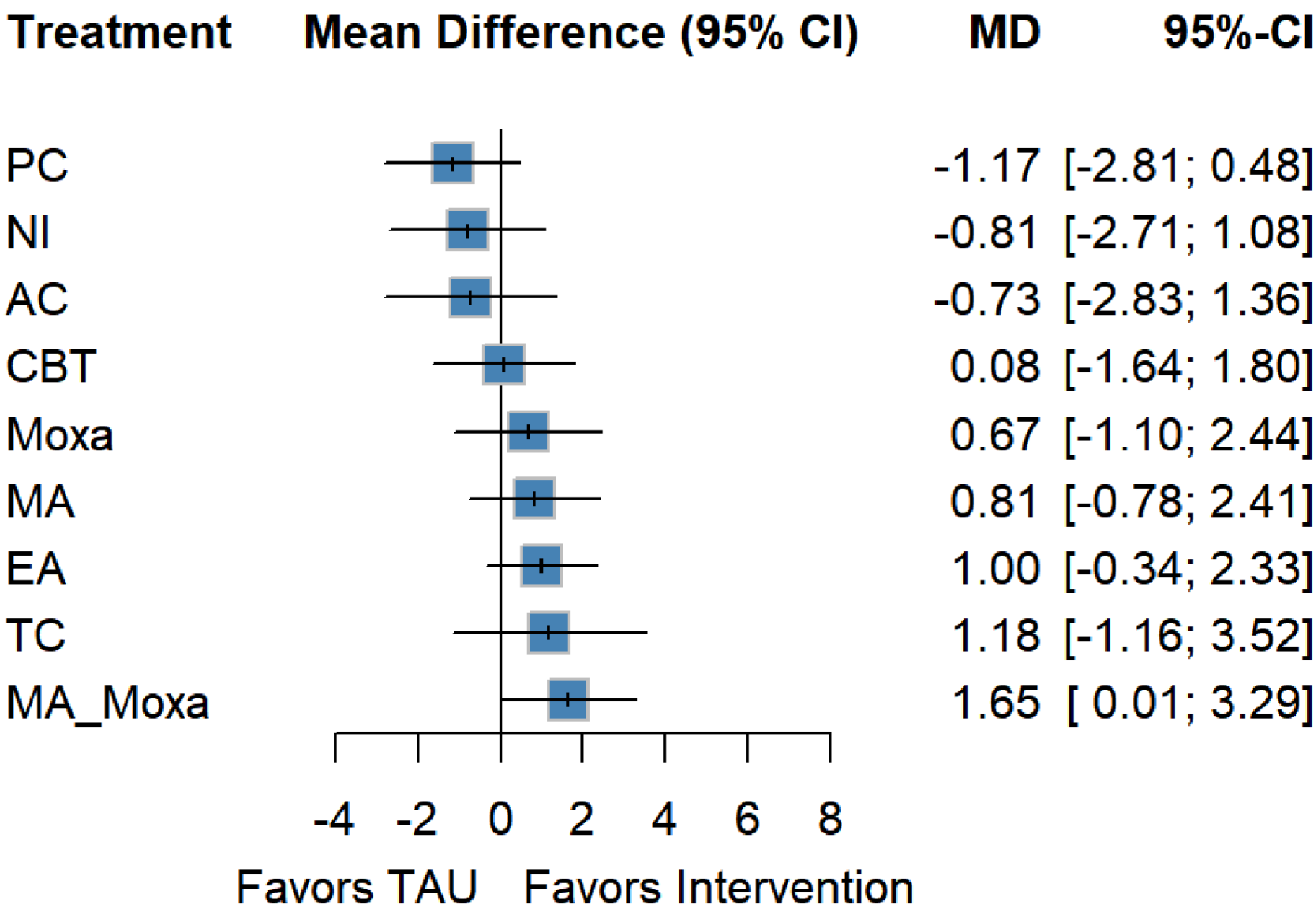

Figure S3. Forest plot for MMSE outcomes.

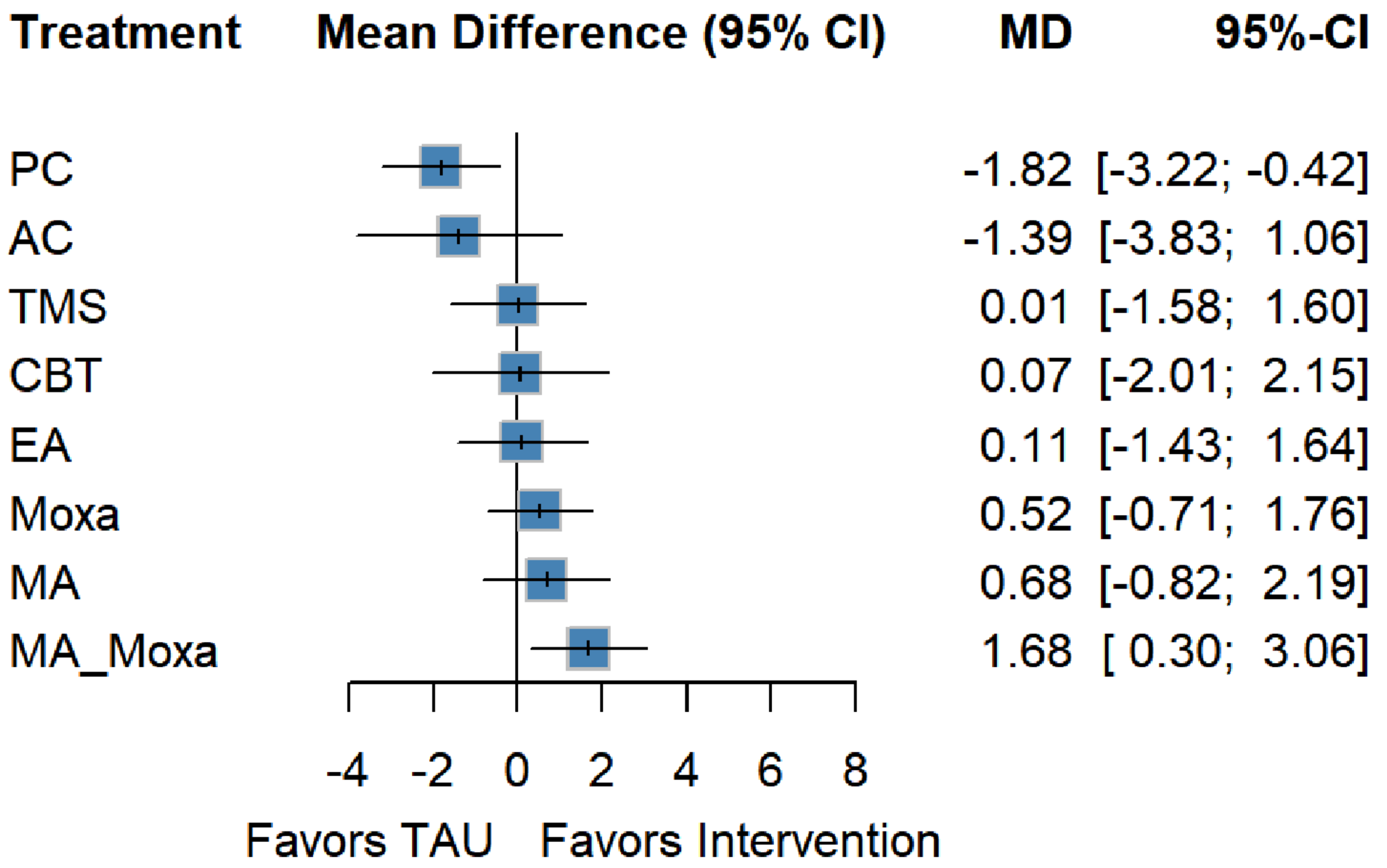

Figure S4. Forest plot for MoCA outcomes.

CINeMA confidence ratings — MMSE

Comparison	No. of studies	Within-study bias	Reporting bias	Indirectness	Imprecision	Heterogeneity	Incoherence	Confidence rating
Mixed evidence								
AC vs CBT	2	No concerns	Some concerns	No concerns	Some concerns	No concerns	No concerns	Moderate
AC vs NI	1	No concerns	Some concerns	No concerns	No concerns	Major concerns	Major concerns	Very low
AC vs TC	2	No concerns	Some concerns	No concerns	No concerns	Some concerns	No concerns	Moderate
CBT vs NI	3	No concerns	Some concerns	No concerns	No concerns	Some concerns	No concerns	Moderate
CBT vs PC	1	Some concerns	Some concerns	No concerns	Some concerns	No concerns	No concerns	Moderate
CBT vs TAU	1	No concerns	Some concerns	No concerns	No concerns	Major concerns	Major concerns	Very low
EA vs TAU	2	No concerns	Some concerns	No concerns	Some concerns	No concerns	No concerns	Moderate
MA vs NI	1	No concerns	Some concerns	No concerns	Some concerns	No concerns	Some concerns	Moderate
MA vs PC	4	No concerns	Some concerns	No concerns	No concerns	Some concerns	Some concerns	Moderate
MA vs TAU	1	Some concerns	Some concerns	No concerns	Some concerns	No concerns	No concerns	Moderate
MA_Moxa vs Moxa	1	Some concerns	Some concerns	No concerns	Some concerns	No concerns	Major concerns	Very low
MA_Moxa vs TAU	1	Some concerns	Some concerns	No concerns	No concerns	Some concerns	Major concerns	Very low
Moxa vs PC	3	Some concerns	Some concerns	No concerns	No concerns	Some concerns	No concerns	Moderate
NI vs TC	1	Some concerns	Some concerns	No concerns	No concerns	Some concerns	No concerns	Moderate
Indirect evidence								
AC vs EA	0	No concerns	Some concerns	No concerns	Some concerns	No concerns	No concerns	Moderate
AC vs MA	0	No concerns	Some concerns	No concerns	Some concerns	No concerns	No concerns	Moderate
AC vs MA_Moxa	0	No concerns	Some concerns	No concerns	Some concerns	No concerns	No concerns	Moderate
AC vs Moxa	0	No concerns	Some concerns	No concerns	Some concerns	No concerns	No concerns	Moderate
AC vs PC	0	No concerns	Some concerns	No concerns	Some concerns	Some concerns	No concerns	Moderate
AC vs TAU	0	No concerns	Some concerns	No concerns	Some concerns	Some concerns	No concerns	Moderate
CBT vs EA	0	No concerns	Some concerns	No concerns	Some concerns	Some concerns	No concerns	Moderate
CBT vs MA	0	No concerns	Some concerns	No concerns	Some concerns	No concerns	No concerns	Moderate
CBT vs MA_Moxa	0	Some concerns	Some concerns	No concerns	Some concerns	No concerns	No concerns	Moderate
CBT vs Moxa	0	Some concerns	Some concerns	No concerns	Some concerns	Some concerns	No concerns	Moderate
CBT vs TC	0	No concerns	Some concerns	No concerns	Some concerns	No concerns	No concerns	Moderate
EA vs MA	0	No concerns	Some concerns	No concerns	Some concerns	Some concerns	No concerns	Moderate
EA vs MA_Moxa	0	Some concerns	Some concerns	No concerns	Some concerns	Some concerns	No concerns	Moderate
EA vs Moxa	0	Some concerns	Some concerns	No concerns	Some concerns	Some concerns	No concerns	Moderate
EA vs NI	0	No concerns	Some concerns	No concerns	Some concerns	No concerns	No concerns	Moderate
EA vs PC	0	No concerns	Some concerns	No concerns	No concerns	Some concerns	No concerns	Moderate
EA vs TC	0	No concerns	Some concerns	No concerns	Major concerns	No concerns	No concerns	Very low
MA vs MA_Moxa	0	Some concerns	Some concerns	No concerns	Some concerns	Some concerns	No concerns	Moderate
MA vs Moxa	0	Some concerns	Some concerns	No concerns	No concerns	Major concerns	No concerns	Very low
MA vs TC	0	No concerns	Some concerns	No concerns	Some concerns	Some concerns	No concerns	Moderate
MA_Moxa vs NI	0	Some concerns	Some concerns	No concerns	No concerns	Some concerns	No concerns	Moderate
MA_Moxa vs PC	0	Some concerns	Some concerns	No concerns	No concerns	No concerns	No concerns	Moderate
MA_Moxa vs TC	0	Some concerns	Some concerns	No concerns	Major concerns	No concerns	No concerns	Very low
Moxa vs NI	0	Some concerns	Some concerns	No concerns	Some concerns	No concerns	No concerns	Moderate
Moxa vs TAU	0	Some concerns	Some concerns	No concerns	Some concerns	Some concerns	No concerns	Moderate
Moxa vs TC	0	No concerns	Some concerns	No concerns	Some concerns	Some concerns	No concerns	Moderate
NI vs PC	0	No concerns	Some concerns	No concerns	Some concerns	Some concerns	No concerns	Moderate
NI vs TAU	0	No concerns	Some concerns	No concerns	Some concerns	Some concerns	No concerns	Moderate
PC vs TAU	0	Some concerns	Some concerns	No concerns	Some concerns	No concerns	No concerns	Moderate
PC vs TC	0	No concerns	Some concerns	No concerns	No concerns	Some concerns	No concerns	Moderate
TAU vs TC	0	No concerns	Some concerns	No concerns	Some concerns	Some concerns	No concerns	Moderate

Figure S5. CINeMA confidence assessment for MMSE comparisons.

CINeMA confidence ratings — MoCA

Comparison	No. of studies	Within-study bias	Reporting bias	Indirectness	Imprecision	Heterogeneity	Incoherence	Confidence rating
				Mixed evidence				
AC vs Moxa	1	No concerns	Some concerns	No concerns	Some concerns	No concerns	No concerns	**Moderate**
CBT vs TAU	1	No concerns	Some concerns	No concerns	Major concerns	No concerns	No concerns	**Very low**
EA vs TAU	1	No concerns	Some concerns	No concerns	No concerns	Major concerns	No concerns	**Very low**
EA vs TMS	1	No concerns	Some concerns	No concerns	No concerns	Major concerns	No concerns	**Very low**
MA vs PC	5	No concerns	Some concerns	No concerns	No concerns	No concerns	No concerns	**Moderate**
MA vs TAU	1	Some concerns	Some concerns	No concerns	Some concerns	No concerns	No concerns	**Moderate**
MA_Moxa vs Moxa	2	Some concerns	Some concerns	No concerns	Some concerns	No concerns	Major concerns	**Very low**
MA_Moxa vs TAU	1	Some concerns	Some concerns	No concerns	No concerns	Some concerns	Major concerns	**Very low**
Moxa vs PC	3	Some concerns	Some concerns	No concerns	No concerns	No concerns	No concerns	**Moderate**
Moxa vs TAU	1	Some concerns	Some concerns	No concerns	No concerns	Some concerns	No concerns	**Moderate**
PC vs TMS	4	Some concerns	Some concerns	No concerns	No concerns	Some concerns	No concerns	**Moderate**
				Indirect evidence				
AC vs CBT	0	No concerns	Some concerns	No concerns	Some concerns	Some concerns	No concerns	**Moderate**
AC vs EA	0	No concerns	Some concerns	No concerns	Some concerns	Some concerns	No concerns	**Moderate**
AC vs MA	0	No concerns	Some concerns	No concerns	Some concerns	No concerns	No concerns	**Moderate**
AC vs MA_Moxa	0	Some concerns	Some concerns	No concerns	No concerns	Some concerns	No concerns	**Moderate**
AC vs PC	0	No concerns	Some concerns	No concerns	Some concerns	Some concerns	No concerns	**Moderate**
AC vs TAU	0	Some concerns	Some concerns	No concerns	Some concerns	No concerns	No concerns	**Moderate**
AC vs TMS	0	No concerns	Some concerns	No concerns	Some concerns	Some concerns	No concerns	**Moderate**
CBT vs EA	0	No concerns	Some concerns	No concerns	Major concerns	No concerns	No concerns	**Very low**
CBT vs MA	0	No concerns	Some concerns	No concerns	Some concerns	Some concerns	No concerns	**Moderate**
CBT vs MA_Moxa	0	Some concerns	Some concerns	No concerns	Some concerns	No concerns	No concerns	**Moderate**
CBT vs Moxa	0	Some concerns	Some concerns	No concerns	Some concerns	Some concerns	No concerns	**Moderate**
CBT vs PC	0	No concerns	Some concerns	No concerns	Some concerns	No concerns	No concerns	**Moderate**
CBT vs TMS	0	No concerns	Some concerns	No concerns	Major concerns	No concerns	No concerns	**Very low**
EA vs MA	0	No concerns	Some concerns	No concerns	Some concerns	Some concerns	No concerns	**Moderate**
EA vs MA_Moxa	0	Some concerns	Some concerns	No concerns	Some concerns	No concerns	No concerns	**Moderate**
EA vs Moxa	0	Some concerns	Some concerns	No concerns	Some concerns	Some concerns	No concerns	**Moderate**
EA vs PC	0	No concerns	Some concerns	No concerns	No concerns	Some concerns	No concerns	**Moderate**
MA vs MA_Moxa	0	Some concerns	Some concerns	No concerns	Some concerns	No concerns	No concerns	**Moderate**
MA vs Moxa	0	Some concerns	Some concerns	No concerns	No concerns	Major concerns	No concerns	**Very low**
MA vs TMS	0	Some concerns	Some concerns	No concerns	Some concerns	No concerns	No concerns	**Moderate**
MA_Moxa vs PC	0	Some concerns	Some concerns	No concerns	No concerns	No concerns	No concerns	**Moderate**
MA_Moxa vs TMS	0	Some concerns	Some concerns	No concerns	Some concerns	No concerns	No concerns	**Moderate**
Moxa vs TMS	0	Some concerns	Some concerns	No concerns	Some concerns	Some concerns	No concerns	**Moderate**
PC vs TAU	0	Some concerns	Some concerns	No concerns	No concerns	Some concerns	No concerns	**Moderate**
TAU vs TMS	0	Some concerns	Some concerns	No concerns	No concerns	Major concerns	No concerns	**Very low**

Figure S6. CINeMA confidence assessment for MoCA comparisons.

SUPPLEMENTARY TABLES

Table S1. Leave-one-out sensitivity analyses: influential studies and heterogeneity after exclusion.

Outcome	Excluded study	I^2 after exclusion (change)
MMSE (base I^2 = 77.6%)	Tan et al. (2017)	62.9% (−14.7%)
MoCA (base I^2 = 73.8%)	Nousia et al. (2023)	54.6% (−19.2%)
	Tan et al. (2017)	63.3% (−10.5%)
	Long et al. (2018)	57.8% (−16.0%)

Note: Only exclusions producing the largest reductions in heterogeneity are shown. The overall treatment rankings and effect directions remained consistent across all leave-one-out scenarios.

Table S2. Sensitivity analysis excluding the MA_Moxa node: SUCRA rankings and global inconsistency before and after exclusion.

	Full network	MA_Moxa node excluded
**MMSE: SUCRA (%)**		
MA_Moxa	87.9	—
TC	76.7	80.2
EA	71.7	62.7
MA	68.2	78.1
Moxa	62.2	85.7
CBT	46.1	51.5
TAU	39.8	28.2
AC	20.5	23.5
NI	17.2	19.6
PC	9.6	20.5
**MoCA: SUCRA (%)**		
MA_Moxa	95.3	—
MA	71.5	83.6
Moxa	65.9	88.5
EA	51.2	52.8
CBT	50.7	46.7
TMS	46.8	55.8
TAU	45.9	41.2
AC	16.8	25.5
PC	5.6	5.8
**Design-by-treatment test (random-effects weighting)**		
MMSE	$Q = 4.99$, $df = 6$, $p = 0.545$	$Q = 2.46$, $df = 5$, $p = 0.783$
MoCA	$Q = 4.71$, $df = 3$, $p = 0.194$	$Q = 0.99$, $df = 2$, $p = 0.610$

Note: SUCRA = surface under the cumulative ranking curve. The excluded node comprised three trials (Qin and Liu, 2023; Xiao et al., 2023; Zhu et al., 2015).

Table S3. Publication bias assessment.

Test	MMSE	MoCA
Egger's intercept (p-value)	2.79 (<0.001)	2.24 (0.002)
Begg's τ (p-value)	0.38 (0.010)	0.28 (0.080)
Trim-and-fill imputed studies	6	7
Adjusted pooled MD (95% CI)	0.92 (0.37 to 1.46)	1.04 (0.50 to 1.58)
Attenuation after adjustment	31%	36%

Note: MD = mean difference; CI = confidence interval. Positive MD values indicate improvement in cognitive scores, consistent with the sign convention used throughout the manuscript.

SEARCH STRATEGIES

2.1 PubMed (from inception to March 24, 2026)

#	Results	Query
39	95	((((randomized controlled trial[Publication Type]) OR (randomized[Title/Abstract])) OR (placebo[Title/Abstract])) AND ... [Combined search: RCT filter + Non-pharmacological interventions + aMCI])
38	1,178,602	((randomized controlled trial[Publication Type]) OR (randomized[Title/Abstract])) OR (placebo[Title/Abstract])
37	1,044,747	((((((((((((((((((((((((((((((("Complementary Therapies"[Mesh]) OR (...)) ... [Full non-pharmacological interventions query])
36	10,076	"Virtual Reality"[Mesh]
35	9	Interviewing, Motivational[Title/Abstract]
34	3,072	"Motivational Interviewing"[Mesh]
33	4,390	"Phototherapies"[Title/Abstract] OR "Light Therapy"[Title/Abstract] OR "Light Therapies"[Title/Abstract] OR "Therapies, Light"[Title/Abstract] OR "Therapy, Light"[Title/Abstract] OR "Photoradiation Therapy"[Title/Abstract] OR "Blue Light Therapy"[Title/Abstract] OR "Blue Light Therapies"[Title/Abstract] OR "Therapy, Blue Light"[Title/Abstract] OR "Blue-light Therapy"[Title/Abstract] OR "Blue-light Therapies"[Title/Abstract] OR "Therapy, Blue-light"[Title/Abstract] OR "Red Light Phototherapy"[Title/Abstract] OR "Phototherapy, Red Light"[Title/Abstract]
32	63,050	"Phototherapy"[Mesh]
31	11,635	"Cognitive Behavioral Therapies"[Title/Abstract] OR "Therapies, Cognitive Behavioral"[Title/Abstract] OR "Therapy, Cognitive Behavioral"[Title/Abstract] OR "Cognition Therapy"[Title/Abstract] OR "Therapy, Cognitive Behavior"[Title/Abstract] OR "Cognitive Behavior Therapies"[Title/Abstract] OR "Therapies, Cognitive Behavior"[Title/Abstract] OR "Therapy, Cognition"[Title/Abstract] OR "Behavior Therapy, Cognitive"[Title/Abstract] OR "Cognitive Behavior Therapy"[Title/Abstract] OR "Cognitive Psychotherapy"[Title/Abstract] OR "Cognitive Psychotherapies"[Title/Abstract] OR "Psychotherapies, Cognitive"[Title/Abstract] OR "Psychotherapy, Cognitive"[Title/Abstract] OR "Therapy, Cognitive"[Title/Abstract] OR "Cognitive Therapies"[Title/Abstract] OR "Therapies, Cognitive"[Title/Abstract] OR "Cognitive Behaviour Therapy"[Title/Abstract] OR "Behaviour Therapy, Cognitive"[Title/Abstract] OR "Cognitive Behaviour Therapies"[Title/Abstract] OR "Therapies, Cognitive Behaviour"[Title/Abstract] OR "Therapy, Cognitive Behaviour"[Title/Abstract] OR "Cognitive Therapy"[Title/Abstract]
30	43,219	"Cognitive Behavioral Therapy"[Mesh]
29	1,812	Mindfulness Meditation[Title/Abstract] OR Meditation, Mindfulness[Title/Abstract] OR Mindfulness Meditations[Title/Abstract]
28	9,030	"Mindfulness"[Mesh]
27	5,215	"Music Therapy"[Mesh]
26	9,745	Biofeedback[Title/Abstract] OR Biofeedbacks[Title/Abstract] OR Myofeedback[Title/Abstract] OR False Physiological Feedback[Title/Abstract] OR False Physiological Feedbacks[Title/Abstract] OR Psychology Biofeedback[Title/Abstract] OR Psychology Biofeedbacks[Title/Abstract]
25	14,457	"Biofeedback, Psychology"[Mesh]
24	3,036	Psychotherapies[Title/Abstract]
23	237,526	"Psychotherapy"[Mesh]
22	18,351	home-based exercise[Title/Abstract] OR aerobic exercise[Title/Abstract] OR rehabilitation exercise[Title/Abstract]
21	283,335	"Exercise"[Mesh]
20	14,767	Massaging[Title/Abstract] OR massage[Title/Abstract] OR Baduanjin[Title/Abstract] OR Taijiquan[Title/Abstract] OR tui na[Title/Abstract]

#	Results	Query
19	38,938	Acupuncture[Title/Abstract] OR Moxibustion[Title/Abstract] OR Electroacupuncture[Title/Abstract] OR Acupuncture, Ear[Title/Abstract] OR auricular acupressure[Title/Abstract]
18	32,979	"Acupuncture Therapy"[Mesh]
17	27,906	"Medicine, Chinese Traditional"[Mesh]
16	21,008	Percutaneous mastoid electrical stimulator[Title/Abstract] OR PMES[Title/Abstract] OR Neuroelectric Biomimetic Stimulator[Title/Abstract] OR NEBS[Title/Abstract] OR fastigial nucleus[Title/Abstract] OR FN[Title/Abstract] OR Transcutaneous Electrical Acupoint Stimulation[Title/Abstract] OR TEAS[Title/Abstract]
15	30	Electroversion[Title/Abstract] OR Cardiac Electroversion[Title/Abstract] OR Electroversion Therapy[Title/Abstract]
14	16,320	"Electric Countershock"[Mesh]
13	12,226	"Transcutaneous Electric Nerve Stimulation"[Mesh]
12	30	transcutaneous auricular vagus nerve stimulation taVNS[Title/Abstract]
11	3,122	"Vagus Nerve Stimulation"[Mesh]
10	8,769	tDCS[Title/Abstract] OR Cathodal Stimulation Transcranial Direct Current Stimulation[Title/Abstract] OR Repetitive Transcranial Electrical Stimulation[Title/Abstract]
9	6,728	"Transcranial Direct Current Stimulation"[Mesh]
8	25,665	TMS[Title/Abstract] OR Repetitive transcranial magnetic stimulation[Title/Abstract] OR rTMS[Title/Abstract]
7	17,854	"Transcranial Magnetic Stimulation"[Mesh]
6	61,816	Therapeutic Electrical Stimulation[Title/Abstract] OR Electrical Stimulation, Therapeutic[Title/Abstract] OR Stimulation, Therapeutic Electrical[Title/Abstract] OR Therapeutic Electric Stimulation[Title/Abstract] OR Electric Stimulation, Therapeutic[Title/Abstract] OR Stimulation,Therapeutic Electric[Title/Abstract] OR Electrical Stimulation Therapy[Title/Abstract] OR Stimulation Therapy, Electrical[Title/Abstract] OR Therapy, Electrical Stimulation[Title/Abstract] OR Therapy, Electric Stimulation[Title/Abstract] OR Stimulation Therapy, Electric Electrotherapy[Title/Abstract] OR Interferential Current Electrotherapy[Title/Abstract] OR Electrotherapy, Interferential Current[Title/Abstract] OR Electric Stimulation[Title/Abstract] OR Electrical Stimulation[Title/Abstract] OR Electrical Stimulations[Title/Abstract] OR Stimulation, Electrical[Title/Abstract] OR Stimulations, Electrical[Title/Abstract] OR Stimulation, Electric[Title/Abstract] OR Electric Stimulations[Title/Abstract] OR Stimulations, Electric[Title/Abstract]
5	102,749	"Electric Stimulation Therapy"[Mesh]
4	18,871	non-pharmacological[Title/Abstract]
3	24,370	Therapies, Complementary[Title/Abstract] OR Therapy Complementary[Title/Abstract] OR Complementary Medicine[Title/Abstract] OR Medicine, Complementary[Title/Abstract] OR Alternative Medicine[Title/Abstract] OR Medicine, Alternative[Title/Abstract] OR Alternative Therapies[Title/Abstract] OR Therapies, Alternative[Title/Abstract] OR Therapy, Alternative[Title/Abstract]
2	266,415	"Complementary Therapies"[Mesh]
1	4,069	"aMCI"[Title/Abstract] OR "Amnestic Mild Cognitive Impairment"[Title/Abstract] OR "Amnestic MCI"[Title/Abstract]

2.2 Embase (from inception to March 24, 2026)

No.	Results	Query
1	6,771	'aMCI':ab,ti OR 'Amnestic Mild Cognitive Impairment':ab,ti OR 'Amnestic MCI':ab,ti
2	93,841	'alternative medicine'/exp
3	47,651	'alternative therapies':ab,ti OR 'alternative therapy':ab,ti OR 'complementary medicine':ab,ti OR 'complementary therapies':ab,ti OR 'eclectic medicine':ab,ti OR 'eclecticism, historical':ab,ti OR 'historical eclecticism':ab,ti OR 'mental healing':ab,ti OR 'metaphysical mind-body relations':ab,ti OR 'mind body technique':ab,ti OR 'mind body therapies':ab,ti OR 'mind body therapy':ab,ti OR 'mind-body relations':ab,ti OR metaphysics:ab,ti OR 'mind-body relations, metaphysical':ab,ti OR 'mind-body therapies':ab,ti OR 'polarity therapy':ab,ti OR radiaesthesia:ab,ti OR radiesthesia:ab,ti OR 'therapeutic cults':ab,ti OR 'thomsonian medicine':ab,ti OR 'therapies, complementary':ab,ti OR 'therapy, complementary':ab,ti OR 'complementary medicine':ab,ti OR 'medicine, complementary':ab,ti OR 'alternative medicine':ab,ti OR 'medicine, alternative':ab,ti OR 'alternative therapies':ab,ti OR 'therapies, alternative':ab,ti OR 'therapy, alternative':ab,ti OR 'complementary therapies':ab,ti
4	396,896	'electrotherapy'/exp
5	76,265	'Therapeutic Electrical Stimulation':ab,ti OR 'Electrical Stimulation, Therapeutic':ab,ti OR 'Stimulation, Therapeutic Electrical':ab,ti OR 'Therapeutic Electric Stimulation':ab,ti OR 'Electric Stimulation, Therapeutic':ab,ti OR 'Stimulation,Therapeutic Electric':ab,ti OR 'Electrical Stimulation Therapy':ab,ti OR 'Stimulation Therapy, Electrical':ab,ti OR 'Therapy, Electrical Stimulation':ab,ti OR 'Therapy, Electric Stimulation':ab,ti OR 'Stimulation Therapy, Electric Electrotherapy':ab,ti OR 'Interferential Current Electrotherapy':ab,ti OR 'Electrotherapy, Interferential Current':ab,ti OR 'Electric Stimulation':ab,ti OR 'Electrical Stimulation':ab,ti OR 'Electrical Stimulations':ab,ti OR 'Stimulation, Electrical':ab,ti OR 'Stimulations, Electrical':ab,ti OR 'Stimulation, Electric':ab,ti OR 'Electric Stimulations':ab,ti OR 'Stimulations, Electric':ab,ti
6	43,197	'Transcranial Magnetic Stimulation'/exp
7	39,495	'TMS':ab,ti OR 'Repetitive transcranial magnetic stimulation':ab,ti OR 'rTMS':ab,ti
8	17,291	'Transcranial Direct Current Stimulation'/exp
9	14,109	'tDCS':ab,ti OR 'Cathodal Stimulation Transcranial Direct Current Stimulation':ab,ti OR 'Repetitive Transcranial Electrical Stimulation':ab,ti
10	17,150	'Vagus Nerve Stimulation'/exp
11	603	'transcutaneous auricular vagus nerve stimulation taVNS':ab,ti
12	25,947	'tens':ab,ti
13	39	'Electroversion':ab,ti OR 'Cardiac Electroversion':ab,ti OR 'Electroversion Therapy':ab,ti
14	29,598	'Percutaneous mastoid electrical stimulato':ab,ti OR 'PMES':ab,ti OR 'Neuroelectric Biomimetic Stimulator':ab,ti OR 'NEBS':ab,ti OR 'fastigii nucleus':ab,ti OR 'FN':ab,ti OR 'Transcutaneous Electrical Acupoint Stimulation':ab,ti OR 'TEAS':ab,ti OR 'Percutaneous mastoid electrical stimulator':ab,ti OR 'PMES':ab,ti
15	100,037	'chinese medicine'/exp
16	70,111	'acupuncture'/exp
17	55,011	'Acupuncture':ab,ti OR 'Moxibustion':ab,ti OR 'Electroacupuncture':ab,ti OR 'Acupuncture, Ear':ab,ti OR 'auricular acupressure':ab,ti
18	21,773	'Massaging':ab,ti OR 'massage':ab,ti OR 'Baduanjin':ab,ti OR 'Taijiquan':ab,ti OR 'tui na':ab,ti
19	573,113	'Exercise'/exp
20	26,790	'home-based exercise':ab,ti OR 'aerobic exercise':ab,ti OR 'rehabilitation exercise':ab,ti

#	Results	Query
21	2,607	'Hyperbaric Oxygenation':ab,ti
22	384,778	'Psychotherapy'/exp
23	4,808	'Psychotherapies':ab,ti
24	12,415	'Biofeedback'/exp
25	14,976	'Biofeedback':ab,ti OR 'Biofeedbacks':ab,ti OR 'Myofeedback':ab,ti OR 'False Physiological Feedback':ab,ti OR 'False Physiological Feedbacks':ab,ti OR 'Psychology Biofeedback':ab,ti OR 'Psychology Biofeedbacks':ab,ti
26	12,186	'Music Therapy'/exp
27	27,145	'Mindfulness'/exp
28	2,696	'Mindfulness Meditation':ab,ti OR 'Meditation, Mindfulness':ab,ti OR 'Mindfulness Meditations':ab,ti
29	49,314	'Cognitive Behavioral Therapy'/exp
30	16,655	'Cognitive Behavioral Therapies':ab,ti OR 'Therapies, Cognitive Behavioral':ab,ti OR 'Therapy, Cognitive Behavioral':ab,ti OR 'Cognition Therapy':ab,ti OR 'Therapy, Cognitive Behavior':ab,ti OR 'Cognitive Behavior Therapies':ab,ti OR 'Therapies, Cognitive Behavior':ab,ti OR 'Therapy, Cognition':ab,ti OR 'Behavior Therapy, Cognitive':ab,ti OR 'Cognitive Behavior Therapy':ab,ti OR 'Cognitive Psychotherapy':ab,ti OR 'Cognitive Psychotherapies':ab,ti OR 'Psychotherapies, Cognitive':ab,ti OR 'Psychotherapy, Cognitive':ab,ti OR 'Therapy, Cognitive':ab,ti OR 'Cognitive Therapies':ab,ti OR 'Therapies, Cognitive':ab,ti OR 'Cognitive Behaviour Therapy':ab,ti OR 'Behaviour Therapy, Cognitive':ab,ti OR 'Cognitive Behaviour Therapies':ab,ti OR 'Therapies, Cognitive Behaviour':ab,ti OR 'Therapy, Cognitive Behaviour':ab,ti OR 'Cognitive Therapy':ab,ti
31	144,280	'Phototherapy'/exp
32	5,749	'Phototherapies':ab,ti OR 'Light Therapy':ab,ti OR 'Light Therapies':ab,ti OR 'Therapies, Light':ab,ti OR 'Therapy, Light':ab,ti OR 'Photoradiation Therapy':ab,ti OR 'Blue Light Therapy':ab,ti OR 'Blue Light Therapies':ab,ti OR 'Therapy, Blue Light':ab,ti OR 'Blue-light Therapy':ab,ti OR 'Blue-light Therapies':ab,ti OR 'Therapy, Blue-light':ab,ti OR 'Red Light Phototherapy':ab,ti OR 'Phototherapy, Red Light':ab,ti
33	10,234	'Motivational Interviewing'/exp
34	21	'Interviewing, Motivational':ab,ti
35	40,990	'Virtual Reality'/exp
36	1,852,772	#02 OR #03 OR ... OR #35 (All intervention terms combined)
37	1,570,134	'randomized controlled trial':ab,ti OR randomized:ab,ti OR placebo:ab,ti
38	172	#01 AND #36 AND #37

2.3 Cochrane Library (from inception to March 24, 2026)

#	Results	Query
1	678	(aMCI):ti,ab,kw OR (Amnestic Mild Cognitive Impairment):ti,ab,kw OR (Amnestic MCI):ti,ab,kw
2	31,306	MeSH descriptor: [Complementary Therapies] explode all trees
3	41,106	(Therapies, Complementary):ti,ab,kw OR (Therapy Complementary):ti,ab,kw OR (Complementary Medicine):ti,ab,kw OR (Medicine, Complementary):ti,ab,kw OR (Alternative Medicine):ti,ab,kw OR (Medicine, Alternative):ti,ab,kw OR (Alternative Therapies):ti,ab,kw OR (Therapies, Alternative):ti,ab,kw OR (Therapy, Alternative):ti,ab,kw
4	69,589	#2 OR #3
5	12,197	MeSH descriptor: [Electric Stimulation Therapy] explode all trees
6	19,954	(Therapeutic Electrical Stimulation):ti,ab,kw OR (Electrical Stimulation Therapy):ti,ab,kw OR (Therapeutic Electric Stimulation):ti,ab,kw OR (Electrotherapy):ti,ab,kw OR (Interferential Current Electrotherapy):ti,ab,kw OR (Electric Stimulation):ti,ab,kw OR (Electrical Stimulation):ti,ab,kw
7	27,018	#5 OR #6
8	3,077	MeSH descriptor: [Transcranial Magnetic Stimulation] explode all trees
9	9,532	(TMS):ti,ab,kw OR (Repetitive transcranial magnetic stimulation):ti,ab,kw OR (rTMS):ti,ab,kw
10	10,206	#8 OR #9
11	2,479	MeSH descriptor: [Transcranial Direct Current Stimulation] explode all trees
12	7,184	(tDCS):ti,ab,kw OR (Cathodal Stimulation Transcranial Direct Current Stimulation):ti,ab,kw OR (Repetitive Transcranial Electrical Stimulation):ti,ab,kw
13	7,717	#11 OR #12
14	399	MeSH descriptor: [Vagus Nerve Stimulation] explode all trees
15	447	(transcutaneous auricular vagus nerve stimulation taVNS):ti,ab,kw
16	708	#14 OR #15
17	3,141	MeSH descriptor: [Transcutaneous Electric Nerve Stimulation] explode all trees
18	1,141	MeSH descriptor: [Electric Countershock] explode all trees
19	1	(Electroversion):ti,ab,kw OR (Cardiac Electroversion):ti,ab,kw OR (Electroversion Therapy):ti,ab,kw
20	1,141	#18 OR #19
21	6,906	(non-pharmacological):ti,ab,kw
22	2,196	(Percutaneous mastoid electrical stimulato):ti,ab,kw OR (PMES):ti,ab,kw OR (Neuroelectric Biomimetic Stimulator):ti,ab,kw OR (NEBS):ti,ab,kw OR (fastigii nucleus):ti,ab,kw OR (FN):ti,ab,kw OR (Transcutaneous Electrical Acupoint Stimulation):ti,ab,kw OR (TEAS):ti,ab,kw OR (Percutaneous mastoid electrical stimulator):ti,ab,kw OR (PMES):ti,ab,kw
23	1,904	MeSH descriptor: [Medicine, Chinese Traditional] explode all trees
24	7,455	MeSH descriptor: [Acupuncture Therapy] explode all trees
25	25,779	(Acupuncture):ti,ab,kw OR (Moxibustion):ti,ab,kw OR (Electroacupuncture):ti,ab,kw OR (Acupuncture, Ear):ti,ab,kw OR (auricular acupressure):ti,ab,kw
26	25,805	#24 OR #25
27	9,983	(Massaging):ti,ab,kw OR (massage):ti,ab,kw OR (Baduanjin):ti,ab,kw OR (Taijiquan):ti,ab,kw OR (tui na):ti,ab,kw
28	773	(Hyperbaric Oxygenation):ti,ab,kw
29	42,398	MeSH descriptor: [Exercise] explode all trees

#	Results	Query
30	46,304	(home-based exercise):ti,ab,kw OR (aerobic exercise):ti,ab,kw OR (rehabilitation exercise):ti,ab,kw
31	77,681	#29 OR #30
32	38,531	MeSH descriptor: [Psychotherapy] explode all trees
33	777	(Psychotherapies):ti,ab,kw
34	38,953	#32 OR #33
35	2,362	MeSH descriptor: [Biofeedback, Psychology] explode all trees
36	5,255	(Biofeedback):ti,ab,kw OR (Biofeedbacks):ti,ab,kw OR (Myofeedback):ti,ab,kw OR (False Physiological Feedback):ti,ab,kw OR (False Physiological Feedbacks):ti,ab,kw OR (Psychology Biofeedback):ti,ab,kw OR (Psychology Biofeedbacks):ti,ab,kw
37	6,022	#35 OR #36
38	1,522	MeSH descriptor: [Music Therapy] explode all trees
39	2,789	MeSH descriptor: [Mindfulness] explode all trees
40	2,906	(Mindfulness Meditation):ti,ab,kw OR (Meditation, Mindfulness):ti,ab,kw OR (Mindfulness Meditations):ti,ab,kw
41	5,064	#39 OR #40
42	15,500	MeSH descriptor: [Cognitive Behavioral Therapy] explode all trees
43	97,466	(Cognitive Behavioral Therapy):ti,ab,kw OR (Cognitive Behavioural Therapy):ti,ab,kw OR (Cognitive Therapy):ti,ab,kw OR (Cognitive Psychotherapy):ti,ab,kw OR (Behavior Therapy):ti,ab,kw OR (Cognition Therapy):ti,ab,kw
44	99,485	#42 OR #43
45	5,316	MeSH descriptor: [Phototherapy] explode all trees
46	16,920	(Phototherapy):ti,ab,kw OR (Light Therapy):ti,ab,kw OR (Photoradiation Therapy):ti,ab,kw OR (Blue Light Therapy):ti,ab,kw OR (Red Light Phototherapy):ti,ab,kw
47	18,277	#45 OR #46
48	1,497	MeSH descriptor: [Motivational Interviewing] explode all trees
49	5,630	(Interviewing, Motivational):ti,ab,kw
50	5,630	#48 OR #49
51	1,749	MeSH descriptor: [Virtual Reality] explode all trees
52	317,686	#4 OR #7 OR #10 OR #13 OR #16 OR #17 OR #20 OR #21 OR #22 OR #23 OR #26 OR #27 OR #28 OR #31 OR #34 OR #37 OR #38 OR #41 OR #44 OR #47 OR #50 OR #51
53	388	#52 AND #1

2.4 Web of Science (from inception to March 24, 2026)

#	Results	Query
1	4,469	TS=(aMCI OR Amnestic Mild Cognitive Impairment OR Amnestic MCI)
2	185,946	TS=(Complementary Therapies OR Therapies, Complementary OR Therapy Complementary OR Complementary Medicine OR Medicine, Complementary OR Alternative Medicine OR Medicine, Alternative OR Alternative Therapies OR Therapies, Alternative OR Therapy, Alternative)
3	15,201	TS=(non-pharmacological)
4	90,844	TS=(Electric Stimulation Therapy OR Therapeutic Electrical Stimulation OR Electrical Stimulation, Therapeutic OR Stimulation, Therapeutic Electrical OR Therapeutic Electric Stimulation OR Electric Stimulation, Therapeutic OR Stimulation,Therapeutic Electric OR Electrical Stimulation Therapy OR Stimulation Therapy, Electrical OR Therapy, Electrical Stimulation OR Therapy, Electric Stimulation OR Stimulation Therapy, Electric Electrotherapy OR Interferential Current Electrotherapy OR Electrotherapy, Interferential Current OR Electric Stimulation OR Electrical Stimulation OR Electrical Stimulations OR Stimulation, Electrical OR Stimulations, Electrical OR Stimulation, Electric OR Electric Stimulations OR Stimulations, Electric)
5	46,796	TS=(Transcranial Magnetic Stimulation OR TMS OR Repetitive transcranial magnetic stimulation OR rTMS)
6	12,324	TS=(Transcranial Direct Current Stimulation OR tDCS OR Cathodal Stimulation Transcranial Direct Current Stimulation OR Repetitive Transcranial Electrical Stimulation)
7	9,885	TS=(Vagus Nerve Stimulation OR transcutaneous auricular vagus nerve stimulation taVNS)
8	7,530	TS=(Transcutaneous Electric Nerve Stimulation)
9	211	TS=(Electric Countershock OR Electroversion OR Cardiac Electroversion OR Electroversion Therapy)
10	108,583	TS=(Percutaneous mastoid electrical stimulato PMES Neuroelectric Biomimetic Stimulator OR NEBS OR fastigii nucleus OR FN OR Transcutaneous Electrical Acupoint Stimulation OR TEAS OR Percutaneous mastoid electrical stimulator OR PMES)
11	56,304	TS=(Medicine, Chinese Traditional)
12	29,051	TS=(Acupuncture Therapy OR Acupuncture OR Moxibustion OR Electroacupuncture OR Acupuncture, Ear OR auricular acupressure)
13	11,229	TS=(Massaging OR massage OR Baduanjin OR Taijiquan OR tui na)
14	482,035	TS=(Exercise OR home-based exercise OR aerobic exercise OR rehabilitation exercise)
15	29,264	TS=(Psychotherapy OR Psychotherapies)
16	9,159	TS=(Biofeedback, Psychology OR Biofeedback OR Biofeedbacks OR Myofeedback OR False Physiological Feedback OR False Physiological Feedbacks OR Psychology Biofeedback OR Psychology Biofeedbacks)
17	5,623	TS=(Music Therapy)
18	15,645	TS=(Mindfulness OR Mindfulness Meditation OR Meditation, Mindfulness OR Mindfulness Meditations)
19	90,435	TS=(Cognitive Behavioral Therapy OR Cognitive Behavioral Therapies OR Therapies, Cognitive Behavioral OR Therapy, Cognitive Behavioral OR Cognition Therapy OR Therapy, Cognitive Behavior OR Cognitive Behavior Therapies OR Therapies, Cognitive Behavior OR Therapy, Cognition OR Behavior Therapy, Cognitive OR Cognitive Behavior Therapy OR Cognitive Psychotherapy OR Cognitive Psychotherapies OR Psychotherapies, Cognitive OR Psychotherapy, Cognitive OR Therapy, Cognitive OR Cognitive Therapies OR Therapies, Cognitive OR Cognitive Behaviour Therapy OR Behaviour Therapy, Cognitive OR Cognitive Behaviour Therapies OR Therapies, Cognitive Behaviour OR Therapy, Cognitive Behaviour OR Cognitive Therapy)
20	92,103	TS=(Phototherapy OR Phototherapies OR Light Therapy OR Light Therapies OR Therapies, Light OR Therapy, Light OR Photoradiation Therapy OR Blue Light Therapy OR Blue Light Therapies OR Therapy, Blue Light OR Blue-light Therapy OR Blue-light Therapies OR Therapy, Blue-light OR Red Light Phototherapy OR Phototherapy, Red Light)

#	Results	Query
21	6,600	`TS=(Motivational Interviewing OR Interviewing, Motivational)`
22	47,139	`TS=(Virtual Reality)`
23	1,234,764	`#2 OR #3 OR #4 OR #5 OR #6 OR #7 OR #8 OR #9 OR #10 OR #11 OR #12 OR #13 OR #14 OR #15 OR #16 OR #17 OR #18 OR #19 OR #20 OR #21 OR #22`
24	1,219,741	`TS=(randomized controlled trial OR randomized OR placebo)`
25	118	`#1 AND #23 AND #24`

2.5 Chinese Databases (CNKI, VIP, Wanfang) — from inception to March 25, 2026

Table S4. Search strategies for the Chinese databases (CNKI, VIP, and Wanfang).

Database	Results	Query
CNKI	45	`(SU="amnestic mild cognitive impairment" OR SU="aMCI" OR SU="forgetful mild cognitive impairment") AND (SU="randomized controlled trial" OR SU="RCT" OR SU="random allocation")`
VIP	36	`(T="amnestic mild cognitive impairment" OR T="aMCI") AND (A="randomized controlled trial" OR A="RCT")`
Wanfang	126	`Title:("amnestic mild cognitive impairment" OR "aMCI") AND Abstract:("randomized controlled trial" OR "RCT" OR "random allocation" OR "randomly assigned")`

** Due to the limited number of relevant Chinese literature, only the target population and study design were used as search criteria to ensure comprehensive retrieval. Further screening was conducted through full-text review.*
